# Mobile money and branchless banking regulations affecting cash-in, cash-out networks in low- and middle-income countries

**DOI:** 10.12688/gatesopenres.12876.1

**Published:** 2018-11-28

**Authors:** Travis W. Reynolds, Marieka Klawitter, Pierre E. Biscaye, C. Leigh Anderson

**Affiliations:** 1 Community Development and Applied Economics, University of Vermont, Burlington, VT, 05405, USA; 2 Daniel J. Evans School of Public Policy and Governance, University of Washington, Seattle, Seattle, WA, 98195, USA; 3 College of Natural Resources, University of California, Berkeley, Berkeley, CA, 94720, USA

**Keywords:** Cash-in cash-out, CICO, Regulation, Policy, Financial inclusion, Digital financial services (DFS), Mobile money

## Abstract

**Background:** We examined recent trends in mobile money and branchless banking regulations related to cash-in, cash-out (CICO) networks (physical access points allowing users to exchange physical cash and electronic money) in low- and middle-income countries, and reviewed evidence on the impacts of CICO regulations on markets and financial inclusion.

**Methods: **Regulation and literature searches began in August 2017 and concluded in June 2018. For the regulatory search we compiled an original database of regulations targeting CICO networks in Bangladesh, India, Indonesia, Kenya, Nigeria, Pakistan, Tanzania, and Uganda. To review evidence of impacts of regulations we conducted additional global searches on Scopus, Google Scholar, and Google using keywords for specific regulatory approaches (e.g., regulation of CICO agents) or hypothesized impacts (e.g., financial inclusion).

**Results:** The resulting database of CICO regulations in the eight focus countries includes 127 regulatory documents, which we coded for four groups of regulations, namely: Business Channel Requirements; Agent Requirements; Regulations on Caps, Fees and Charges; and Customer Identification Requirements. Early CICO regulations focused on agent selection rules, limits on fees, and know-your-customer requirements. More recent waves of regulation have expanded or restricted services CICO agents provide, and also imposed reporting requirements on service providers in an effort to prevent fraud or enhance financial inclusion. Our search for evidence of impacts of CICO regulations resulted in a sample of 90 documents published since 2005, of which only 31 provided evidence on CICO regulation impacts, with most limited in scope—suggesting rigorous policy analysis remains lacking in this quickly expanding sector.

**Conclusions:** Many low- and middle-income countries have introduced regulations that may affect CICO networks, with regulatory approaches differing across geographies and over time. While anecdotal reports of regulatory impacts exist, we found limited evidence of impacts of regulations on CICO networks or on CICO-related financial inclusion.

## Introduction

Only an estimated 62% of adults worldwide have a bank account through a formal financial institution, leaving over 2 billion adults unbanked ( Demirgüç–Kunt *et al.*, 2015). The financial infrastructure gap is greatest in many low- and middle-income countries (LMICs), which continue to be characterized by very low numbers of bank branches (e.g., 2.9 branches per 100,000 people in Ethiopia versus 13.5 in India and 32.9 in the U.S.) and limited access to automated teller machines (ATMs) (e.g., 0.5 ATMs per 100,000 people in Ethiopia versus 19.7 in India and 173 in the U.S.) ( Beck *et al.*, 2007; World Bank, 2015a; World Bank, 2015b). While conventional banks have long struggled to extend their networks into low-income and rural communities ( Chaia *et al.*, 2009), digital financial services (DFS) have offered the potential to extend financial opportunities to these previously under-served populations ( Radcliffe & Voorhies, 2012). However the use of DFS requires consumer access to cash-in, cash-out (CICO) networks—physical access points including bank branches but also including “branchless banking” ^1^ options such as ATMs, point-of-sale (POS) terminals, agents ^2^, and cash merchants – in order to be able to convert physical cash to electronic money. For rural and low-income populations far from physical bank infrastructure, CICO networks may provide much-needed access to financial services such as bank accounts or loans, as well as money transfer options. Broadening CICO networks may thus extend financial opportunities for low-income and rural populations by increasing the availability of branchless access points in those communities, including via local retailers and other trusted intermediaries that partner with more remote banks or mobile network operators (MNOs) ( Lyman *et al.*, 2006; Maurer *et al.*, 2013; Radcliffe & Voorhies, 2012). Multiple authors have argued that CICO networks could help fill the financial infrastructure gap in low-income countries and increase financial inclusion ( Alexandre, 2011; Andrianaivo & Kpodar, 2011; Ivatury & Mas, 2008; McKay & Pickens, 2010).

Few studies have reported on the overall impacts of expanding CICO networks, but many report positive impacts of branchless banking and mobile money. Pénicaud & Katakam (2013) find that mobile money has already extended payment and other financial services in many developing countries, with more mobile money accounts than bank accounts in nine countries (Cameroon, the Democratic Republic of Congo, Gabon, Kenya, Madagascar, Tanzania, Uganda, Zambia, and Zimbabwe). They add that MNO agents providing CICO services are more prevalent than bank branches in 44 countries, and that this has contributed to the uptake in mobile money ( Pénicaud & Katakam, 2013), which is viewed as safer, cheaper, and easier to deliver than physical cash ( Mas & Sullivan, 2012). McKay & Pickens (2010) observe that “branchless banking prices to consumers are already marginally lower than comparable services and will likely fall as branchless banking matures” (p. 12).

The expansion of DFS through retail agents, either led by banks or nonbank commercial actors such as MNOs, has also exhibited the potential to extend financial services to unbanked and marginalized communities ( Lyman *et al.*, 2006), offering lower transaction costs and greater accessibility ( Villasenor *et al.*, 2016). According to the Global System for Mobile Communications Association (GSMA) (2015), approximately half of the global population that is financially excluded has access to a mobile phone, creating an opportunity for mobile money, digital credit, and other DFS to reduce the financial access gap. For many low-income customers, mobile financial services will provide their first access to any formal financial services (e.g., checking and savings accounts or loans), and these formal services can be safer, cheaper, and less time consuming than informal financial alternatives (e.g., borrowing or transferring money through relatives, friends, money lenders, and traders) ( Andrianaivo & Kpodar, 2011; Kashuliza *et al.*, 1998; Lyman *et al.*, 2006). By facilitating the exchange between cash and electronic money, CICO networks may thus increase financial inclusion opportunities through further reducing barriers to the adoption of mobile money and other forms of DFS ( Radcliffe & Voorhies, 2012; Villasenor *et al.*, 2016).

As mobile money and branchless banking expand, countries are developing new regulations to govern DFS operations ( Dolan, 2009; Gutierrez & Singh, 2013; Ivatury & Mas, 2008; Lyman *et al.*, 2006; Mas, 2011), including regulations targeting aspects of the different CICO interfaces. These regulations, especially those targeting agents—individuals who provide access to mobile money and branchless banking services—may restrict the potential for CICO networks to expand, and may affect, positively or negatively, the potential for CICO networks to support financial inclusion. While the expansion of DFS has shown the potential to increase financial inclusion ( Radcliffe & Voorhies, 2012; Villasenor *et al.*, 2016), CICO physical access points remain necessary for the exchange between physical cash and mobile money ( Mas & Sullivan, 2012). This report summarizes types of recent mobile money and branchless banking regulations related to CICO networks in eight low- and middle-income countries and reviews available evidence on the impacts these regulations may have on CICO markets and consumers.

## Methods

### Initial approach

For this review, we first searched for regulations that may specifically affect agent-based CICO networks and then for evidence of impact of these regulations on CICO network expansion, functioning, and financial inclusion. All searches began in August 2017 and were concluded by June 2018.

### Review of regulations governing CICO networks in eight low- and middle-income countries

For the regulatory search, we compiled an original database of regulations potentially affecting CICO networks based on reports from Central Banks or other financial authorities in eight countries in sub-Saharan Africa and South and Southeast Asia: Bangladesh, India, Indonesia, Kenya, Nigeria, Pakistan, Tanzania, and Uganda. According to data from the 2015 Financial Inclusion Insights survey ( Intermedia, 2015), levels of financial inclusion vary widely across these countries (summarized in Table 1). While 66.1% of the population in India has a formal bank account, for example, only 8.7% in Pakistan and 9.4% in Tanzania engages in formal banking. Among those who have heard of mobile money, 80.2% have adopted (ever used) mobile money in Kenya, compared to only 5.1% in India and 5.5% in Indonesia. These countries may therefore serve as illustrative case studies of different levels of mobile money network development and the CICO networks that facilitate the exchange between physical cash and digital mobile money ( Macmillan *et al.*, 2016).

**Table 1.** Summary of regulations affecting cash-in, cash-out (CICO) networks in eight selected countries.

Country	% adoption of traditional and digital financial services, 2017 ^4^	Mobile money agent outlets registered per 100,000 adults, 2015	(ATMs per 100,000 adults, 2015) ^5^	Business channel requirements		Agent requirements			Restrictions on fees and charges		Customer identification requirements ^6^
				Can banks use agents for CICO?	Can non- banks use agents for CICO?	Use of exclusive agents	Who is excluded from being an agent (individual or institution types)	Pre-existing period of business requirement for agent	Can agents charge clients additional fees (i.e. fees to the agent)?	Caps on account balance or transactions (see Table 2 for more detail)	
Bangladesh	39	541.35	6.85	Yes	Only if partner with commercial bank	Super- agent ^7^ allowed; Retail agent required	Loan defaulters; convicts	Not specified	No, bank shall pay a reasonable fee/ commission to agents	Yes, on transactions; differ by account. type and transaction type	Two factor identification: PIN/ Biometric scan for all transactions
India	44	NA	19.70	Yes	Only on behalf of a bank	Allowed	Non-Bank Financial Institutions (2010), restrictions removed 2014		Not specified	Yes, on transfer	PIN for all trans- actions, “officially valid document” or simplified norms for “small accounts” ^8^ when opening an account
Indonesia	47	NA	53.31	Yes	Only business with remitter’s license for cash-out	Required for bank agents	Not specified	At least two years old for small banks and MNO agents	No	Yes, on transactions and account balance	Government issued ID, driver’s license or passport when opening an account
Kenya	78	519.54	9.81	Yes	Yes	Prohibited	Faith- based, NGOs, NPOs, educational institutions; foreign exchange bureaus	Continuous business permit at least 18 months before application	No	Yes. E-Money: transaction limit, monthly load limit	Two factor identification: IDs, PINs, passwords, ATM, secret codes/ messages for all transactions
Nigeria	53	20.82	16.20	Yes	Yes	Prohibited	Faith- based, nonprofits, NGOs, educational institutions, currency exchanges	At least 1 year for both agents and super agents ^9^	No	Limit on account balance and daily transactions differ by acct. type	IDs when opening an account, PINS, passwords, payment card, secret code or secret message for all transactions
Pakistan	36	245.26	8.77	Yes	Only if with financial institution	Allowed	Not specified	Super agent needs to be well- established	No, fees are decided by financial institutions (FIs); agents share revenue with FIs	Yes, on transfer and account balance	ID, mobile number, purpose of transactions ^10^
Tanzania	42	917.62	6.00	Yes	Yes	Prohibited	Those without other business activities ^11^	At least two years before application	No	Yes, on transfer and account balance	ID and mobile number for non-bank personal account CICO ^12^
Uganda	58	526.65	4.44	Yes	Only if partner with financial institution	Prohibited	Not a business or without physical address	Not specified	No, agents earn commission from providers	Yes, provider required to set limits on frequency and volume	ID and PIN for transactions

**References:** Bangladesh Bank, 2011a; Bangladesh Bank, 2011b; Bangladesh Bank, 2013a; Bangladesh Bank, 2013b; Bangladesh Bank, 2015; Bangladesh Bank, 2017; Bangladesh Telecommunication Regulatory Commission, 2004; Bank Indonesia, 2009a; Bank Indonesia, 2009b; Bank Indonesia, 2017; Bank of Tanzania, 2007; Bank of Tanzania, 2013; Bank of Tanzania, 2015a; Bank of Tanzania, 2015b; Bank of Tanzania, 2017; Bank of Uganda, 2013; Bank of Uganda, 2016; Bank of Uganda, 2017; Central Bank of Kenya, 2010; Central Bank of Kenya, 2013a; Central Bank of Kenya, 2013b; Central Bank of Kenya, 2013c; Central Bank of Kenya, 2014; Central Bank of Nigeria, 2007; Central Bank of Nigeria, 2012; Central Bank of Nigeria, 2013a; Central Bank of Nigeria, 2013b; Central Bank of Nigeria, 2014a; Central Bank of Nigeria, 2014b; Central Bank of Nigeria, 2014c; Central Bank of Nigeria, 2015a; Central Bank of Nigeria, 2015b; Central Bank of Nigeria, 2015c; Central Bank of Nigeria, 2015d; Central Bank of Nigeria, 2015e; Central Bank of Nigeria, 2016; Central Bank of Nigeria, 2017; CGAP, 2010a; Hidayati, 2011; Indonesia Financial Services Authority, 2014; KPMG Indonesia, 2015; Macmillian *et al.*, 2016; Pakistan Telecommunication Authority, 2016; Pakistan Telecommunication Authority, 2017; Reserve Bank of India, 2010; Reserve Bank of India, 2012; Reserve Bank of India, 2014a; Reserve Bank of India, 2014b; Reserve Bank of India, 2014c; Reserve Bank of India, 2014d; Reserve Bank of India, 2014e; Reserve Bank of India, 2016a; Reserve Bank of India, 2016b; Reserve Bank of India, 2016c; Reserve Bank of India, 2016d; Staschen, 2015; State Bank of Pakistan, 2016a; State Bank of Pakistan, 2016b; State Bank of Pakistan, 2016c; State Bank of Pakistan, 2016d; Tanzania Revenue Authority, 2014; Telecom Regulatory Authority of India, 2012; Telecom Regulatory Authority of India, 2013; Telecom Regulatory Authority of India, 2016.

**Table 2.** Broad patterns in timing of cash-in, cash-out (CICO) regulations.

Types of regulation common in initial regulations	Types of regulation more typical of later regulations
1. *Agent selection* (regulations that determine whether banks and non-banks may use agents, and that set minimal requirements to become an agent)	1. *Agent services* (the designation of classes between agents (e.g., sub-agents) and what services they may provide)
2. *Caps and fees* (limits on account or transaction sizes and on the fees that CICO providers may charge to customers)	2. *Reporting requirements* (reporting of agent characteristics (e.g., gender, rural vs. urban) and the processes for consumers to authenticate agents (e.g., agent has logo of their financial institution stickered to the window))
3. *Reporting requirements* (reporting agent and CICO locations)	
4. *KYC requirements* (procedures for identifying and verifying the identity of clients)	

Supplementary File 1 provides a list of search strings used to identify regulations, conducting systematic searches in Google. We also reviewed regulatory documents from relevant national government websites, including Central Banks, Telecom Regulators and Competition Authorities, among others. The resulting database consists of 127 regulatory documents, including the text of regulations themselves (54), peer-reviewed articles describing provisions within specific regulations (13), or grey literature sources (60) describing specific regulations that might relate to CICO networks in each target country. We systematically coded regulations for the presence of provisions that may affect CICO networks in the following areas:

- *Business channel requirements*, including various rules requiring agent authentication procedures and reporting of CICO locations (Reporting Requirements), and rules surrounding interoperability across financial services providers (Interoperability);
- *Agent requirements*, including rules surrounding who may be an agent (Agent Selection) and what services agents may provide (Agent Services);
- *Caps, fees and charges regulation*, including limits on account or transaction sizes, and limits on fees charged by providers; and
- *Customer identification requirements*, including Know-Your-Customer (KYC) rules for verifying the identity of clients.

**Figure 1.**
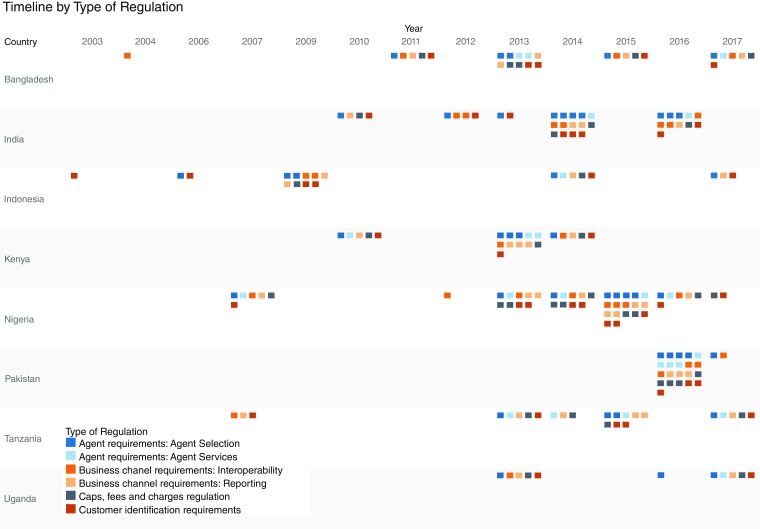
Timeline of cash-in, cash-out (CICO) regulations by country and regulation type.

A summary of regulations potentially affecting CICO networks in Bangladesh, India, Indonesia, Kenya, Nigeria, Pakistan, Tanzania, and Uganda, by provision and by country over time, is provided in Figure 1 (data available from Reynolds *et al*., 2018). The full database of regulatory documents collected and coded is provided in Supplementary File 2. Supplementary File 3 provides a typology of regulatory decision options, highlighting differences in the choice of regulatory approaches across the eight study countries, and Supplementary File 4 provides more detailed descriptions of the regulatory context within each country.

### Review of evidence of regulatory impacts on CICO networks

To search for evidence of impacts on markets and consumers of mobile money and branchless banking regulations potentially affecting CICO networks, we did not limit our search by geography ^3^. We targeted reports that discuss the regulation of CICO networks, mobile money, branchless banking, or DFS more broadly. We conducted searches on Scopus, Google Scholar, and Google using 16 search strings ( Supplementary File 1), some modified by adding specific terms for target organizations. We then conducted supplemental searches using additional targeted keywords: “agent exclusivity”; “agent network expansion” and “CICO network expansion” (further described in Supplementary File 5 and Supplementary File 6).

Preliminary searches returned a total of 1,643,889 results, of which 2,023 abstracts were reviewed together with searching the full text of each document for key words. We narrowed this initial pool to 90 CICO-related articles based on the following criteria: each article (i) made reference to CICO networks in some form; (ii) was published after 2005; and (iii) was publicly available in English. We classified these 90 documents into three categories of relevance relating to our research question around the impacts of regulations on CICO networks:

- *Directly relevant* documents both 1) describe CICO-related regulation(s) and 2) discuss the impact(s) of the regulation(s).
- *Indirectly relevant* documents describe CICO-related regulation(s), but do not discuss impact(s).
- *Secondary* documents discuss CICO networks but not regulation(s) or impact(s).

Of the 90 documents in the sample, we considered 31 to be “directly relevant.” We did not identify any documents mentioning regulation of “cash-in, cash-out networks” specifically, but many discuss particular mobile money and branchless banking regulations that affect CICO networks. Of the 31 “directly relevant” articles, 26 discuss regulation of both mobile money and branchless banking or agents, 4 only discuss regulations related to mobile money, and 2 only discuss regulations related to ATMs. We also included information from 10 additional “indirectly relevant” documents in the Results, as these articles provide additional context on CICO-related regulations and reported impacts.

Of the 31 “directly relevant” articles that contain evidence of impact, 22 present empirical or anecdotal evidence but do not test for associations between regulations and impacts, and the remainder (9 articles) document predicted evidence. Articles that provide what we refer to as “anecdotal” evidence describe associations between a specific regulation in a specific country and a particular outcome (e.g., increased adoption of mobile wallets in India from less stringent KYC requirements), but do not test these associations. Articles that provide estimates of predicted impacts describe potential impacts that a regulation may have on a country or population, but do not test or evaluate the potential impacts nor the assumptions behind their predictions.

## Results

### Trends in regulations targeting CICO networks

Table 1 summarizes the regulations we identified targeting CICO networks in Bangladesh, India, Indonesia, Kenya, Nigeria, Pakistan, Tanzania and Uganda. Some regulations have the potential to limit the growth of CICO networks, for example limits on the use of agents for CICO transactions by non-banks (such as MNOs that offer mobile money services), requirements for agent exclusivity (meaning agents cannot provide services for multiple financial service providers), requirements for agents to have business licenses, and agent location restrictions. Other regulations may have a mix of positive and negative effects on the growth of CICO markets depending on context and the passage of time: for example, Macmillan *et al.* (2016) state that regulations such as customer identification requirements may initially limit the growth of CICO services, but may also help protect against fraud which could otherwise undermine CICO network expansion. Tarazi & Breloff (2011), meanwhile, predict that some regulations such as agent exclusivity may encourage early CICO market growth, but later limit competition in the market for CICO services (examined further in Supplementary File 5).

Figure 1 illustrates the evolution of CICO regulations by country and regulation type (Business channel requirements, Agent requirements, Caps, fees and charges regulation, and Customer identification requirements, including Know-Your-Customer (KYC) rules).

We observe no consistent “pathway” that CICO regulatory regimes follow across countries over time. Rather, regulations targeting CICO networks often come in clusters targeting multiple aspects of CICO systems (rather than piecemeal regulatory approaches building over time). Few countries change their regulations (i.e., revise a given type of regulation targeting CICO networks once that regulation has been established) over time.

### Trends in business channel requirements

Several common regulations target banks and other financial services providers, with rules surrounding who may serve as a CICO agent and what requirements must be met (including reporting) to engage in CICO market activities. All countries surveyed require that banks authenticate CICO agents both during registration and on an ongoing basis. Requirements on authentication during registration typically state that a bank must have clear due diligence procedures, and an agent must pass these procedures. Seven countries (Bangladesh, Indonesia, Kenya, Nigeria, Pakistan, Tanzania, Uganda) further require banks to report on information related to the physical location of agents, including information such as an agent’s physical address and telephone number. Regulations in Tanzania ( Bank of Tanzania, 2013), Nigeria ( Central Bank of Nigeria, 2013b) and Bangladesh ( Bangladesh Bank, 2017) require that this information be published. Of the eight countries, six (Bangladesh, Indonesia, Kenya, Nigeria, Tanzania, Uganda) require banks to regularly submit information to regulators on agent banking activities, including information such as the value and volume of transactions.

All eight countries also currently have some regulatory structures surrounding interoperability of CICO services. The timing of interoperability regulations, however, varied widely from country to country. In Bangladesh, Nigeria, Pakistan, Tanzania, and Uganda, platform or agent interoperability regulations were included among the first rounds of regulations. In India, Indonesia, and Kenya, interoperability policies were only included in later regulations.

Uganda and Kenya have had platform interoperability mandates—requiring CICO service providers and agents to use similar/interoperable software platforms—since 2013, but no account interoperability mandates (i.e., no requirements that service providers allow access to accounts held by other service providers, even if technically possible). As a result, mobile money providers may still refuse to interoperate with other providers, or if they do allow access across a range of providers, may set lower prices for transfers within their own network than transfers across different networks. Macmillan *et al.* (2016) find that because of the lack of an account interoperability mandate, new mobile money providers in Uganda are finding it difficult to enter the market. Similarly, Bourreau & Valetti, 2015 assert that progress towards interoperability in Kenya may not have come about without government action. In Kenya, Safaricom opened its network of agents to Airtel in 2014, just before the Competition Authority of Kenya ordered Safaricom to open up its network of agents to rivals ( Bourreau & Valetti, 2015). As of December 2017, Safaricom continues to have over 69% of the market share for mobile subscriptions ( Communications Authority of Kenya, 2018).

Four countries (Bangladesh, India, Nigeria, Pakistan) require that financial service platforms and mobile money service providers utilize systems that are platform- and account- interoperable with other payment systems in the country. In all four countries, platform interoperability mandates consistently appeared before account interoperability mandates. In Indonesia and Tanzania, interoperability regulations exist but do not clearly distinguish between platform- and account-interoperability.

### Trends in agent requirements

For regulations on who may become a CICO agent, Kenya ( Central Bank of Kenya, 2010), Nigeria ( Central Bank of Nigeria, 2013b), and Tanzania ( Bank of Tanzania, 2013) have required that agents have an established commercial activity that has been active for a specified period of time prior to engaging in agent banking. Similarly, Bangladesh requires agents to have sound financial capacity and has a minimum employee requirement ( Bangladesh Bank, 2013a; Bangladesh Bank, 2013b), and since 2014, Indonesia has required that agents have had a viable source of income for two years prior to conducting agent banking ( Asian Development Bank, 2017). Kenya ( Central Bank of Kenya, 2010) and Tanzania ( Bank of Tanzania, 2013) uniquely require that agents have more than one job (beyond agent banking). The most commonly reason stated in regulations for excluding an agent are if the proposed agent is a current or recent employee of the financial institution conducting the banking (Bangladesh, Kenya, Uganda), or if the proposed agent operates out of a not-for-profit institution (Kenya and Nigeria).

Regulations in India and Indonesia further include location requirements for agents. The 2014 Laku Pandai pilot program in Indonesia required agents to be domiciled in the program location ( Asian Development Bank, 2017). In India, 25% of payment access points are required to be in rural centers ( Reserve Bank of India, 2014a).

Regulations surrounding Agent Services (i.e., what activities CICO agents are or are not allowed to undertake) vary markedly across regions. For example, among the four African countries in our focus, agent exclusivity has been explicitly disallowed (since 2013 in Nigeria, Tanzania, and Uganda, and since 2010 for Kenya; i.e., agents must be allowed to work for more than one financial institution or mobile service provider). In contrast, in the four South Asian countries, agent exclusivity is either mandated (so agents are only allowed work for one bank or service provider (Bangladesh, Indonesia)); partially mandated where certain types of agents can only provide services for one bank (India); or financial institutions are free to practice agent-exclusivity if they so choose (Pakistan). In the case of Bangladesh, early regulation only mandated exclusivity for retail agents and sub-agents, but the most recent regulation ( Bangladesh Bank, 2017) mandates exclusivity for all agents. Conversely, recent regulations in Pakistan ( State Bank of Pakistan, 2016a; State Bank of Pakistan, 2016b) allow agents to provide services for more than one bank if agreements are reached with each, but do not prohibit exclusivity.

Four countries (Kenya, Pakistan, Tanzania, Bangladesh) also have regulations related to e-float or cash holdings maintained by agents. Bangladesh, Kenya and Pakistan require that agents either have a specified fixed deposit amount or credit limit, or show they sufficient have sufficient funds to cover operations. Conversely, Tanzania specifies a maximum daily balance (float) that agents are permitted to hold ( Bank of Tanzania, 2015b).

### Trends in caps, fees and charges regulation

Pakistan, Nigeria and Bangladesh have caps on account balances and transactions that vary by account type. The most commonly regulated caps on CICO activity are on the frequency, volume, and/or value of transactions by customers. For example, Indonesia’s 2009 E-Money regulation states that the largest electronic money value limit for a registered type is Rp 5,000,000 (five million rupiah) ( Bank Indonesia, 2009b).

Six countries restrict CICO agents from charging customers any fee beyond the financial institution’s prescribed fees (Bangladesh, India, Indonesia, Kenya, Nigeria, Tanzania, Uganda). Policy in Pakistan simply notes that charge and fee sharing structures must be agreed upon beforehand in the contract between the financial institution and the agent ( State Bank of Pakistan, 2016d).

### Trends in KYC regulation

We observe different regional patterns for CICO Know Your Customer (KYC) regulations in South Asian compared to African countries. Among South Asian countries in the sample, KYC requirements have moved from general KYC requirements that are similar to traditional banks, to KYC requirements that are customized for the type of account and/or applicant. In Bangladesh, a 2015 regulation simplified KYC requirements for mobile accounts exclusively engaging in low-value transactions ( Bangladesh Bank, 2015). Regulations in Indonesia ( Bank Indonesia, 2017) and India ( Reserve Bank of India, 2014d) stipulate that people who lack an “officially valid document” can still open a bank account with a photograph and reference letter from a local community member or government official if the bank deems them “low risk.” In India, these simplified KYC requirements for opening accounts also apply to “small account” transactions at Payment Banks. In Pakistan, customers face different requirements based on the level of account sought. Generally, lower-level accounts are for individuals engaging in basic transactions while high-level accounts are for individuals as well as joint accounts, firms, trusts, and businesses. Correspondingly, a level zero account requires a verified SIM card, national identity card, and photo; a level one account additionally requires a biometric and cell phone number; and a level two account requires further verification regarding Anti-Money Laundering and Combating the Financing of Terrorism laws ( State Bank of Pakistan, 2016a).

In Bangladesh, KYC requirements were initially limited and then became increasingly stringent over time for certain accounts. To open an account in 2011, consumers were only required to file a KYC Profile (i.e., a form turned into the bank that requires a customer address and signature) ( Bangladesh Bank, 2011a). But in 2015, Bangladesh began to require two-step verification ( Bangladesh Bank, 2015), and in 2017 verification required a National ID ( Bangladesh Bank, 2017). Similarly, to conduct a transaction in 2011, only two-factor authentication was required, but additional requirements for a verified National ID and fingerprints were then added in 2013 ( Bangladesh Bank, 2013b). (However, current regulations still stipulate that mobile accounts utilized for low value transactions should be subject to risk-proportionate, simplified KYC procedures.)

In contrast, Nigeria initially had very stringent KYC requirements for conducting transactions and then became less stringent over time. In 2007, Point of Service (POS) scanners were required to be updated to include fingerprint reader/scanners ( Central Bank of Nigeria, 2007). Regulations allowing for differing KYC requirements for different account levels were introduced in 2013 ( Central Bank of Nigeria, 2013a); however, regulation in 2017 further relaxed KYC requirements for the lowest-level accounts ( Central Bank of Nigeria, 2017). In 2015, Nigeria relaxed overall KYC requirements and now only requires a PIN and mobile number to conduct a transaction ( Central Bank of Nigeria, 2015b; Central Bank of Nigeria, 2015c).

Nigeria is the only country among the African countries reviewed that also customizes KYC requirements by the level of the account. For example in Kenya, payment service providers require identity card numbers or passport numbers from all customers to open an account ( Central Bank of Kenya, 2014).

Table 1 summarizes broad patterns in the relative timing of CICO regulations by type in the countries in our sample (with the caveat that there is no single pathway followed by all countries in developing regulations around CICO networks, and some regulations, such as the timing of interoperability regulations, followed no discernable pattern. Supplementary File 3 provides a typology of regulatory decision options, highlighting differences in the choice of regulatory approaches across the eight study countries. Supplementary File 4 provides more detailed descriptions of the regulatory context within each country.

### Evidence of impact of CICO regulations

We next report findings from the 31 “directly relevant” articles which contain evidence of impacts of regulations affecting CICO networks. We use information from 10 “indirectly relevant” articles detailing CICO regulatory requirements but not their impacts to provide additional context on the types of regulations affecting CICO networks. We first report evidence from two studies that review general impacts of CICO regulations in the Overall Impact of CICO Regulations section. The sections that follow are broken out by Business Channel Requirements, Agent Requirements, Restrictions on Fees and Charges, and Customer Identification Requirements, which follow the categories of regulations identified in our background review of regulations affecting CICO networks in eight countries.

### Overall impact of CICO regulations

There are two studies ( Asian Development Bank, 2017; Evans & Pirchio, 2015) that report generally on impacts of regulations affecting CICO networks, as opposed to discussing specific types of regulations. In the first report, the Asian Development Bank (ADB) studies and quantifies the role digital finance can play in accelerating financial inclusion, including through expanding CICO networks, in Indonesia, the Philippines, Cambodia, and Myanmar (2017). Findings are derived from more than 80 interviews with stakeholders in each country, supported by extensive secondary research and economic analysis. The study recommends specific regulatory measures to support digital finance and enable financial inclusion, including real-time KYC, digitization of the credit process, and digitally enabled agents and applications. The ADB then calculates the estimated impact of their recommended regulatory measures in each country, looking at predicted increases in electronic payment flows, additional credit uptake, savings mobilization, GDP, and incomes for populations earning less than $3 per day. Predicted impacts vary by country: for example, there is a predicted $2 billion increase in electronic payment flows in Cambodia compared to $7 billion in the Philippines and $50 billion in Indonesia. The report does not distinguish impacts for suggested regulatory measures that would specifically affect CICO networks. The study does discuss impacts of specific existing regulations, however, and these impacts are included in the Business Channel Requirements, Agent Requirements, Account Restrictions, and Identification Requirements sections below.

In the second report, Evans & Pirchio (2015) identify 22 developing countries (14 in Africa, 5 in Asia, and 3 in Latin America) in which mobile money schemes have been attempted and evaluate whether mobile money has succeeded or failed in each country. The study considers the transfer of electronic money and CICO services via agents as relevant mobile money platforms. The authors analyze information on the percentage of adults with a mobile money account, the percent of adults that have used their mobile money account recently, the percent of adults that have used mobile money through an agent or through a separate, non-mobile money account, and the proportion of mobile money transactions per GDP. Based on these measures, the authors categorize each country into one of four classifications: mobile money ignited with explosive growth (Bangladesh, Cote d’Ivoire, Kenya, Rwanda, Somaliland, Tanzania, Uganda, and Zimbabwe); mobile money ignited with slow growth (Ghana, Pakistan, and the Philippines); mobile money failed (Burkina Faso, Haiti, India, Indonesia, Madagascar, Mexico, Nigeria, and South Africa); and too early or not enough data available to determine the growth of mobile money (Democratic Republic of Congo, Paraguay, and Sri Lanka).

Evans & Pirchio (2015) then explore each country’s market structure, products offered, and the regulatory framework to evaluate characteristics that may contribute to a successful or failed mobile money market. Of these qualitative characteristics, the authors find that regulatory frameworks are the most important factor contributing to the success or failure of mobile money in each country. They consider whether specific mobile money regulations exist, whether non-banks can issue mobile money, and whether there are KYC requirements in each country. The authors define “heavy regulatory environments” as regulatory frameworks that require banks to play a central role in mobile money, have burdensome KYC requirements, and place restrictions on agents. Restrictions on agents are the primary type of regulation included in the review that affects CICO networks: countries that have failed to ignite mobile money markets typically have restrictions on who can operate as an agent (through restrictions on whether banks or non-banks can contract services out to agents), restricting mobile money companies’ ability to bring a critical mass of agents on board ( Evans & Pirchio, 2015).

The report concludes that these heavy regulatory environments generally contribute to failed mobile money schemes ( Evans & Pirchio, 2015). Of the eight countries that experienced explosive mobile money growth, seven have light regulatory environments with minimal limitations and restrictions on mobile money and allow non-banks to issue mobile money. The only country that has a heavy regulatory environment but that still experienced explosive mobile money growth is Bangladesh, which requires mobile financial services to be bank-led. Evans & Pirchio (2015) attribute the growth in Bangladesh to a large network of bank agents who customers use primarily for paying bills. But with the exception of the success of mobile money in Bangladesh, the report concludes that almost all countries with heavy regulations and specific mandates for bank-led mobile money models have failed to ignite the mobile money market. The authors do not, however, test for associations between regulatory characteristics and mobile money outcomes.

### Impacts of business channel requirements

Business channel requirements are regulations that specify the institutions that may provide financial services, including CICO services. These regulations are indirectly connected to CICO networks in that they may affect the growth or spread of mobile money networks, or branchless banking, and through this affect the growth of CICO networks. We found 27 documents (out of the 41 “directly relevant” or “indirectly relevant” documents reviewed) that discuss regulations or regulatory impacts pertaining to business channel requirements.

Five papers discuss regulations affecting liability for the provision of financial services. In Brazil, India, Kenya, and South Africa, banks are fully liable for agents who deliver financial services ( Akhter & Khalily, 2017; Lyman *et al.*, 2006; Prochaska & Brix, 2008; Tarazi & Breloff, 2010). Tazari & Breloff (2010) note that in Kenya, MNOs such as Safaricom are not liable for the actions of agents, although banks with CICO access points are liable for their agents. In Tanzania, Nigeria, and Liberia, banks are responsible for overseeing all non-bank mobile money services and are responsible for approving non-bank entities before they can provide mobile money services ( Makulilo, 2015). None of these studies report on the impacts of these regulations.

Fourteen documents discuss regulations targeting the ability of non-banks and MNOs to provide financial and CICO services. A 2016 regulation in Myanmar allowed MNOs and non-banks to offer DFS without limitations or a requirement to partner with banks; however, Myanmar is unique in this regard ( Asian Development Bank, 2017). In Bangladesh, Pakistan, India, and Tanzania, MNOs are required to partner with banks in order to deliver mobile money services, including CICO services ( CGAP, 2010c; Di Castri & Gidvani, 2014; Makulilo, 2015; Sultana, 2014). The European Investment Bank (2014) states that in Mozambique there is no partnership requirement, however MNOs are required to register as a non-bank financial institution in order to deliver DFS. Similarly, in Indonesia MNOs are required to obtain a remitter license in order to provide cash-out services ( Bourreau & Valetti, 2015; CGAP, 2010a; Hidayati, 2011). Mohammad (2015) also notes that in Indonesia MNOs can only partner with registered entities for the provision of DFS, including CICO services. MNOs are prohibited from providing cash-out services in Bangladesh ( Parvez *et al.*, 2015). Lyman *et al.* (2006) state that in South Africa, non-banks are restricted from issuing e-money. While non-banks are permitted to issue e-money in Kenya and Indonesia, in Kenya MNOs are required to store e-money deposits in a financial institution ( Gupta, 2016), and in Indonesia non-banks are required to have a minimum of two years of business experience in order to issue e-money ( USAID, 2015).

Of the 31 documents directly discussing impacts of regulations, six pertain to business channel requirements. These regulations target both bank networks and non-bank financial services such as MNOs and other e-money providers. There are four studies reporting on the negative impacts of these business channel requirement regulations, providing anecdotal evidence from Indonesia ( Asian Development Bank, 2017), Bangladesh ( Parvez *et al.*, 2015), Cameroon ( European Investment Bank, 2014), the Central African Republic ( *ibid.*), and India ( Sultana, 2014). These studies find that regulations which limit the ability of MNOs to provide DFS reduce the products and services available to customers (including CICO services), negatively impact financial inclusion, and limit growth of the market as a whole.

Similarly, four studies provide anecdotal evidence from Myanmar ( Asian Development Bank, 2017), Sri Lanka ( Sultana, 2014), Pakistan ( Sultana, 2014), and the Philippines ( Di Castri, 2013) on the positive impacts of allowing MNOs to provide mobile money services with fewer restrictions. These positive impacts include: extending services to unbanked populations; larger markets; and improved cost, quality, and variety of services, including CICO services. Two studies note that the majority of the fastest growing mobile money markets in the world are in countries that allow MNO deployments, with mobile money accounts surpassing bank accounts in Kenya, Madagascar, Tanzania, and Uganda ( Di Castri, 2013; Sultana, 2014). Additionally, Di Castri (2013) describes that in the Philippines, the central bank released regulation that allowed MNOs to compete with banks to deliver mobile money services, and that “competition [to banks] from MNO-based remittances has not only enriched the variety of services available, it has also been an important driver in lowering the price of remittances” (p. 14).

### Impacts of agent requirements

Agent requirements are regulations that govern the entities that interact directly with customers and provide CICO services, and include regulations that specify what organizations can have agents and who can be an agent. These requirements are directly connected to CICO networks as these agents are often directly responsible for providing CICO services. We found 35 documents (out of 41 “directly relevant” or “indirectly relevant” documents) that describe regulations related to agent requirements, including 26 discussing regulatory impacts. Of these 26 documents, 3 speculate on impact and 23 provide anecdotal evidence of impact.

In many countries, including Brazil, Bangladesh, Pakistan, India, Sri Lanka, Cameroon, Nigeria, Benin, Mozambique, Senegal, Zambia, Mexico, South Africa, Indonesia, and Liberia, banks are permitted to use agents for CICO services ( Akhter & Khalily, 2017; Diniz *et al.*, 2014; European Investment Bank, 2014; GIZ NABARD Rural Financial Institutions Programme, 2014; Gupta, 2016; Lyman *et al.*, 2006; Makulilo, 2015; Mohammad, 2015; Parvez *et al.*, 2015; Sultana, 2014; USAID, 2015). In Indonesia, the Laku Pandai regulations passed in 2014 specifically allow banks to use agents for branchless banking services ( Asian Development Bank, 2017). In contrast, the Bank of Uganda Mobile Money Guidelines 2013 state that Ugandan banks are prohibited from using agent networks for CICO services, except through official partnerships with MNOs ( European Investment Bank, 2014; Makulilo, 2015).

In addition to banks using (or being prohibited from using) agents for CICO services, many countries allow non-banks to use agents for CICO services, including Indonesia, Kenya, the Philippines, Tanzania, Benin, Cameroon, Mozambique, Nigeria, Senegal, Uganda, Zambia, Liberia, Sri Lanka, and Sudan ( Alampay, 2010; Bourreau & Valetti, 2015; CGAP, 2010b; Di Castri & Gidvani, 2014; European Investment Bank, 2014; Evans & Pirchio, 2015; Gupta, 2016; Karrar & Rahman, 2015; Lyman *et al.*, 2006; Makulilo, 2015; Maurer *et al.*, 2013; Muthiora, 2015; Sultana, 2014). However, Cambodia prohibits non-banks from using agents ( Asian Development Bank, 2017).

Regulations in India, Tanzania, Indonesia, Uganda, Brazil, and Pakistan limit who can be an agent, often based on the host organization’s size (e.g., how many outlets a company has) and type (e.g., only postal offices can operate as agents) ( CGAP, 2010c; Di Castri & Gidvani, 2014; European Investment Bank, 2014; Jayo *et al.*, 2012; Lyman *et al.*, 2006; Muthiora, 2015; Prochaska & Brix, 2008).

Many countries have minimum requirements for e-float or cash holdings including Kenya, Indonesia, Bangladesh, Sri Lanka, Afghanistan, the Philippines, Cambodia, Malaysia, Pakistan, Tanzania, and India ( CGAP, 2010a; CGAP, 2010c; Claessens & Rojas-Suarez, 2016; Di Castri, 2013; Jansen, 2010; Parvez *et al.*, 2015; Stapleton, 2013; Tarazi & Breloff, 2010). Most often these cash holding requirements are proportionately based on the total amount of money held in deposit, however in Tanzania constant, minimum thresholds are specified in the regulation ( Di Castri & Gidvani 2014).

Anecdotal evidence on the impacts of agent requirements on CICO networks was presented in nine reports. Two studies in Indonesia ( Claessens & Rojas-Suarez, 2016; Mohammad, 2015) report that regulations limiting the type of agent (i.e., registered vs. unregistered entities) have a negative impact on the number of agents operating in low-income and rural communities, as well as the number of mobile money users. Two additional studies in Indonesia ( Di Castri, 2013; Hidayati, 2011) report that regulations limiting the type of services agents can provide (i.e., cash-in vs. cash-out) discourage smaller agents from entering the market and have a negative impact on the number of mobile money users and transactions. Two studies from the Philippines ( Asian Development Bank, 2017; Di Castri, 2013) report anecdotal evidence that regulations limiting the number of agents have limited the growth of the mobile money market. One study in Brazil ( Diniz *et al.*, 2014) reports that regulations allowing a variety of retail outlets to act as agents drastically increased the number of mobile banking points from fewer than 15,000 in 2000 to over 150,000 in 2010. CGAP reports (2010a) that Bank Indonesia intended to promote the use of formal remittance channels with a new regulation, but unintentionally hindered the development of these channels by requiring that agents be licensed as money remitters.

Several studies report anecdotal evidence on agent interoperability ^13^. Agent interoperability determines whether agents exclusively provide CICO services for a single MNO or bank, or whether agents can provide CICO services for multiple platforms ( Bourreau & Valetti, 2015). In Kenya and India, banks are prohibited from establishing exclusive contracts with agents ( CGAP, 2010b; Gupta, 2016; Klein & Mayer, 2011; Kulkarni, 2015; Muthiora, 2015). One study reports that regulations prohibiting agent exclusivity in Tanzania incentivized three leading MNOs to establish an interoperability agreement in June 2014 ( Bourreau & Valetti, 2015). This agreement allows customers to send and receive mobile money through any of the MNOs involved in the agreement ( *Ibid.*). Two studies ( Gupta, 2016; Muthiora, 2015) predict that 2011 and 2014 regulations prohibiting agent exclusivity in Kenya may begin to promote interoperability. Bourreau & Valetti (2015) anecdotally report that Safaricom allowed a rival service to use its agent network in anticipation of the 2011 and 2014 regulations.

Jansen (2010) observes that banks in Kenya face additional rules that regulate the bank-agent relationship from the 2010 Banking Agent Guidelines issued by the Central Bank of Kenya, while MNO-agent relationships remain comparatively unregulated. For example, banks must choose agents from a specific list of registered businesses that have been operating for at least two years, and then the Central Bank of Kenya must approve each agent. Additionally, banks must participate in a shared agent network and cannot establish exclusive agent contracts. Comparing this with M-PESA’s experience, to become an agent businesses must submit an initial deposit of USD$1,300, provide a bank statement with six months of cash flow, and sign an exclusive contract; however, these actions are company protocol and not mandated by government regulations. Jansen reports that banks may have access to a higher quality pool of agents as a result of the Guidelines, but that the requirements may limit their ability to develop a stable agent network that can compete with M-PESA. These Banking Agent Guidelines were designed to emulate the bank-led Brazilian branchless banking system and allow banks to use agents for CICO services, just as M-PESA uses agents. Jansen further reports that Brazil established their branchless banking model in 2003 when banks only had branches in 1,500 of the country’s 5,500 municipalities. By 2010, a system of 80,000 agents with POS terminals were active in all 5,500 municipalities. This agent system created an estimated seven million new customer accounts in Brazil. Jansen predicts but does not test whether this model of branchless banking will succeed in Kenya. Since Safaricom dominates the mobile money market, the behavior of Safaricom will likely affect whether banks can successfully operate in the mobile money market. Safaricom can refuse to share their agent network, or cooperate with banks for agent interoperability, with more positive predicted impacts on the number of new customers accessing financial services if Safaricom shares their agent network with banks.

### Impacts of restrictions accounts, transactions, fees and charges

Requirements on fees and charges include regulations related to caps on transactions, caps on account balances, fees for CICO services, and taxes on CICO services. These regulations may either be directly or indirectly connected to CICO networks. Regulations which involve fees or taxes for CICO services directly affect CICO networks. However, regulations pertaining to mobile money networks or branchless banking, including fees for these services or caps on account balances, indirectly affect CICO networks. Of the 41 documents reviewed, we found 26 that discuss regulations related to fees and charges.

Many countries prohibit agents from charging fees for services (including but not limited to CICO services) additional to those charged by the financial service provider, including in Kenya, India, Indonesia, and Bangladesh ( CGAP, 2010a; CGAP, 2010b; GIZ NABARD Rural Financial Institutions Programme, 2014; Parvez *et al.*, 2015; Prochaska & Brix, 2008), while in Pakistan agents can charge fees if these are first approved by a bank ( CGAP, 2010c). Agents may receive commission in Bangladesh ( Parvez *et al.*, 2015) and India ( GIZ NABARD Rural Financial Institutions Programme, 2014).

Several countries allow banks, MNOs, or non-bank financial institutions to charge fees for CICO services, including Nigeria ( Adam & Awoyemi, 2014), Kenya ( European Investment Bank, 2014; Jansen, 2010), Uganda ( Duncombe, 2012), and the Philippines ( Alampay, 2010). In addition, some countries including Cambodia, the Philippines, Kenya, and Indonesia allow banks and MNOs to charge interbank and inter-entity fees for transfers ( Asian Development Bank, 2017). We also found evidence of one tax on CICO services: in 2013, a 10% excise duty on money transfer services, including CICO networks, was introduced in Kenya ( Muthiora, 2015).

Caps on transactions, including on the amount and frequency of CICO transactions, exist in Bangladesh, the Democratic Republic of Congo, India, Indonesia, Kenya, Peru, the Philippines, South Africa, Nigeria, Namibia, Pakistan, and Sri Lanka ( Akhter & Khalily, 2017; Bangladesh Business News, 2017; CGAP, 2010a; Claessens & Rojas-Suarez, 2016; Di Castri, 2013; Gupta, 2016; Kathuria, 2016; Lyman *et al.*, 2006; Muthiora, 2015; Oluwafemi & Ola, 2014; Sultana, 2014; USAID, 2015). Three studies state that these caps are mandated by a regulation or set by the Central Bank, as in Bangladesh, the Philippines, and India ( Bangladesh Business News, 2017; Lyman *et al.*, 2006), while the others do not make clear whether the caps are set by regulations or determined by the financial service provider. Additionally, we found evidence that caps on account balance amounts exist in Brazil, Cote D’Ivoire, India, Kenya, Peru, Indonesia, and the Philippines ( CGAP, 2010b; Claessens & Rojas-Suarez, 2016; Evans & Pirchio, 2015; Lyman *et al.*, 2006; Muthiora, 2015; Pareek & Raman, 2016; USAID, 2015). One study ( Lyman *et al.*, 2006) states that caps on account balance amounts in the Philippines are set by the Central Bank; the other studies do not make clear who sets the cap.

Out of the 31 documents 5 provide anecdotal evidence of impacts of regulations related to fees and charges. One study ( Asian Development Bank, 2017) suggests that interoperability has been constrained in several countries including Cambodia, the Philippines, Indonesia, and Kenya because interbank and inter-entity transfers are still discouraged, in part due to fees. One study ( Das, 2014) reports that ATM usage grew dramatically in India between 2009 and 2014 due to regulations that made all third-party ATM transactions free. Two studies ( Di Castri & Gidvani, 2014; Muthiora, 2015) describe anecdotal evidence of the impact of taxes on mobile money transactions. Muthiora (2015) describes how the 10% excise duty on money transfer services in Kenya has resulted in higher transaction costs to customers, and suggests that low-income communities may choose informal ways of transferring money in response to the rising costs of basic transactions. Di Castri & Gidvani (2014) state that taxes on mobile money transfers in Tanzania threaten uptake and usage. Finally, in Indonesia a USAID study (2015) provides anecdotal evidence that caps on account balances have had a negative impact on the use of mobile banking for certain services (e.g., loan disbursements, collections).

### Impacts of customer identification requirements

Customer identification requirements include regulations related to Know Your Customer (KYC)/Customer Due Diligence (CDD) and Anti-Money Laundering (AML)/Combating the Financing of Terrorism (CFT) requirements. These regulations are intended to prevent criminal activity such as money laundering, fraud, or funding terrorism ( Evans & Pirchio, 2015). Banks and agents conduct identification processes through actions such as obtaining formal customer identification, verifying customer identity, and assessing the risk of customers. Strict identification requirements could limit the ability of low-income customers to use mobile money and agent-based CICO services ( Di Castri *et al.*, 2015). Of the 41 documents reviewed, 30 discuss regulations related to identification requirements. Of these 30 documents, 17 simply state the existence of identification requirements for financial services or the existence of KYC/CDD and AML/CFT regulations in low- and middle-income countries ( Adam & Awoyemi, 2014; Akhter & Khalily, 2017; Alampay, 2010; Bangladesh Business News, 2017; CGAP, 2010a; CGAP, 2010b; CGAP, 2010c; Claessens & Rojas-Suarez, 2016; Di Castri & Gidvani, 2014; Duncombe, 2012; European Investment Bank, 2014; Gupta, 2016; Jansen, 2010; Kemal & Yan, 2015; Maurer *et al.*, 2013; Muthiora, 2015; Oluwafemi & Ola, 2014).

The Financial Action Task Force (FATF), an organization which sets international AML/CFT standards, encouraged a risk-based approach to AML/CFT requirements in their 2012 recommendations to help pursue financial inclusion ( Basel Committee on Banking Supervision, 2015). We found evidence that many countries have simplified identification requirements associated with low-value accounts. These countries include Pakistan, Sri Lanka, Indonesia, South Africa, Brazil, India, Kenya, Tanzania, Mexico, Peru, and Fiji ( Asian Development Bank, 2017; Di Castri, 2013; Kathuria, 2016; Lyman *et al.*, 2006; Sultana, 2014; USAID, 2015). While in many countries some form of identification is still required for account opening, in India, Fiji, Tanzania ( Di Castri, 2013; Lyman *et al.*, 2006) some banks allow customers to provide alternatives to formal identification to verify their identity, such as a letter from a public official. In India ( Kathuria, 2016) there are no KYC/CDD requirements for e-wallets up to INR 10,000 (USD$150). In some countries, including Kenya, the Philippines, and Bangladesh, regulations allow for agents to conduct KYC/CDD and AML/CFT procedures ( Klein & Mayer, 2011; Parvez *et al.*, 2015; Prochaska & Brix, 2008). However, in Bangladesh banks are still accountable for ensuring compliance ( Sultana, 2014).

There are four studies reporting anecdotal evidence that regulations allowing for modified identification led to an easier process for opening new mobile money accounts, increased financial inclusion, and larger adoption of mobile money in India ( GIZ NABARD Rural Financial Institutions Programme, 2014; Kathuria, 2016; Sultana, 2014), Sri Lanka ( Di Castri, 2013; Sultana, 2014), and Pakistan ( Di Castri, 2013). Additionally, four studies report anecdotal evidence that strict identification requirements led to the exclusion of marginalized populations—such as low-income or migrant workers—from the market, and negatively impacted market growth in many African countries ( Makulilo, 2015) including South Africa ( Lyman *et al.*, 2006) as well as in Indonesia ( Stapleton, 2013) and the Philippines ( Prochaska & Brix, 2008).

## Discussion

Many LMICs have introduced regulations that affect CICO networks; however, systematic evidence of the impacts of these regulations is limited. While we found anecdotal reports, we did not identify any studies formally testing the impact of regulations affecting CICO networks. Some hypotheses follow common wisdom: making access more difficult or costly is likely to reduce growth. What is missing, however, is evidence on the magnitude of any negative consequences which can then be compared against the positive outcomes intended by the regulatory measure. Without evidence of the regulatory tradeoffs, of which we found none, one is left speculating on whether, for example, the benefits of reduced fraud outweigh the costs of customer identification requirements, and importantly, how the benefits and costs are distributed across sub-populations within a country, including the poorest and most remote.

Of the anecdotal evidence, some studies report that “heavy” regulatory environments can constrain the growth of mobile money networks ( Evans & Pirchio, 2015). For example, regulations that prohibit or limit MNOs from providing mobile money services can have a negative impact on the CICO services available to unbanked populations ( European Investment Bank, 2014; Parvez *et al.*, 2015; Sultana, 2014). Some predictions are reasonable—that regulations on agents, such as those which limit the type of agent or services agents can provide, can reduce the number of agents in rural or low-income communities ( Claessens & Rojas-Suarez, 2016; Di Castri, 2013; Hidayati, 2011; Mohammad, 2015)—but largely untested ( Supplementary File 5 further describes a selection of recent efforts to regulate the activities of CICO agents via agent exclusivity rules). The interoperability of mobile money technology and agent networks can also impact the use of CICO services. Some evidence suggests that greater interoperability can lead to more customers using mobile money services ( Asian Development Bank, 2017).

Regulations that directly involve customers, such as regulations on fees and identification requirements, may limit the ability of rural or low-income populations to participate in mobile money networks. Fees to open or maintain bank accounts can be prohibitive for low-income individuals ( European Investment Bank, 2014). Additionally, many unbanked individuals are unable to provide appropriate identification to meet KYC/AML requirements ( Lyman *et al.*, 2006; Makulilo, 2015; Prochaska & Brix, 2008; Stapleton, 2013). Relaxing identification and fee requirements, or creating alternative accounts such as basic savings accounts with fewer restrictions, may increase the number of rural and low-income individuals who can participate in formal banking networks ( Asian Development Bank, 2017; Di Castri, 2013; Di Castri & Gidvani, 2014; GIZ, 2014; Kathuria, 2016; Sultana, 2014), though this has not been demonstrated empirically.

Government-sponsored programs that, though not regulations, indicate government support for mobile banking could promote expanded CICO access as described in two studies. A report by the ADB (2017) discusses the TabunganKu basic savings account initiatives from the Central Bank of Indonesia, which aims to increase the number of customers using mobile money and branchless banking services. A total of 12 million TabunganKu mobile savings accounts were opened between 2010 and 2014. Another study ( Kemal & Yan, 2015) describes government-to-person (G2P) payments by the Pakistani government that used mobile banking and agents to deliver cash to transfer recipients. To the extent these government policies expand mobile banking networks, they would also expand the associated CICO networks that facilitate the operation of mobile banking. Alexandre (2011) argues that branchless banking may succeed in broadening financial inclusion in the future, as investments in infrastructure now will reduce future costs, and financial services offered through these institutions may be modified to better target low-income and unbanked populations.

Literature describing the regulatory environment of CICO networks also provides many suggestions for improving regulations. Supplementary File 6 describes a selection of recent efforts that appear directed at expanding CICO networks, particularly among previously under-served rural populations. Stapleton (2013) argues that regulators should find a compromise between strengthening regulations for security and solvency of banking networks, while also easing some regulations to increase access to financial services ( Di Castri & Gidvani, 2014; Lyman *et al.*, 2006; Pareek & Raman, 2016; Sultana, 2014). Makulilo (2015) argues that direct communication between regulators, banks, and MNOs regarding any new regulations will help identify cost-effective ways to implement monitoring and reporting processes. And the Asian Development Bank (ADB) (2017) recommends that regulators should focus on easing supply-side regulatory barriers that limit the availability of financial services. The ADB ultimately concludes that CICO networks could expand if regulators allow businesses to test new ideas, including allowing both collaboration and competition between banks and MNOs.

## Data availability

The data used in Figure 1 and the full database of CICO regulations and literature used in this analysis are available through the Open Science Framework: https://doi.org/10.17605/OSF.IO/QJ3GA ( Reynolds *et al.*, 2018). Data are available under the terms of the Creative Commons Zero "No rights reserved" data waiver (CC0 1.0 Public domain dedication).

## Footnotes

^1^Branchless banking is defined by the Alliance for Financial Inclusion (2017) as the delivery of financial services outside conventional bank branches through agents or other third party intermediaries using technologies such as card-reading point-of-sale (POS) terminals and mobile phones to transmit transaction details. Branchless banking “is not limited to bank services; it also includes an array of financial services provided by nonbanks” (p. 3). Branchless banking may follow bank-based, nonbank-based, or mixed models ( CGAP, 2008).

^2^The GSMA (2010) defines an agent as “a person or business that is contracted to facilitate transactions for users. The most important of these are cash-in and cash-out (i.e., loading value into the mobile money system, and then converting it back out again); in many instances, agents register new customers too. Agents usually earn commissions for performing these services. They also often provide front-line customer service—such as teaching new users how to initiate transactions on their phone. Typically, agents will conduct other kinds of business in addition to mobile money… Some industry participants prefer the terms “merchant” or “retailer” to describe this person or business to avoid certain legal connotations of the term “agent” as it is used in other industries.”

^3^ Supplementary File 1 provides the list of search strings we used to identify regulations and their associated impacts, conducting searches on Scopus, Google Scholar, and Google. We also conducted a series of supplemental searches, modifying these search strings by adding specific terms for target organizations and geographies.

^4^Adoption data are from the 2017 Brookings Financial and Digital Inclusion Project Report ( Lewis *et al.*, 2017)

^5^Mobile money agent outlets and ATMS per 100,000 adults data are from the IMF Financial Access Survey ( International Monetary Fund, 2018)

^6^Customer identification requirements include regulations related to Know Your Customer (KYC)/Customer Due Diligence (CDD) and Anti-Money Laundering (AML)/Combating the Financing of Terrorism (CFT) regulations. These regulations are intended to prevent criminal activity such as money laundering, fraud, or funding terrorism ( Evans & Pirchio, 2015)

^7^Super agents are responsible for managing and controlling subagents

^8^Two factor authentication required for all mobile banking transactions involving debits. Simplified norms only for small accounts, otherwise complete KYC compliance required.

^9^Super agents in Nigeria do not hold e-money at their platform, they monitor and manage their agent networks. Super agents and agents both required to be existing companies operational for at least 12 months prior to obtaining license. Agents need to be “well established”.

^10^In 2015, mobile operators were required to link mobile SIM cards with customers’ biometric data (fingerprints) and national ID. The Central Bank now allows customers with verified SIMs to open accounts remotely. (The original plan used a biometric fingerprint scan at an agent to open the lowest level account, but compliance proved difficult due to high costs of fingerprint readers.)

^11^This is only for being a bank agent. MNOs’ agents do not have to satisfy this requirement

^12^Bank agents in Tanzania are required to use two-factor authentication.

^13^Additional studies report anecdotal evidence on platform interoperability. Platform interoperability refers to the interoperability between mobile money platforms from different financial service providers. In Ghana and Tanzania, interoperability between mobile money platforms is mandated ( Di Castri, 2013; Di Castri & Gidvani, 2014). The Asian Development Bank (2017) reports that in Indonesia, the three largest MNOs developed the first interoperability agreement of its kind in 2013 which has allowed customers to transact with others across networks. The report also finds that in Bangladesh, the B-Kash mobile money network reached 11 million accounts in two and a half years due to network interoperability. Finally, the ADB finds that in the Philippines, the lack of MNO interoperability has limited the growth of e-money use.

## Supplementary material

**Supplementary File 1. Summary of search strings used to compile literature on cash-in, cash-out (CICO) regulations.**

**Supplementary File 2. Coding spreadsheet of cash-in, cash-out (CICO) network regulations.**

This file contains the full literature database used in the development of this article.

**Supplementary File 3. Typology of cash-in, cash-out (CICO) regulatory decision options.**

This file contains a summary typology of regulatory decision options pursued by different countries to govern different aspects of CICO networks.

**Supplementary File 4. Cash-in, cash-out (CICO) regulation summaries by country.**

This file contains a detailed summary of regulatory approaches surrounding CICO networks in eight countries: Bangladesh, India, Indonesia, Kenya, Nigeria, Pakistan, Tanzania, and Uganda.

**Supplementary File 5. Agent exclusivity regulations and market growth.**

This file contains the results of supplemental targeted searches surrounding CICO agent exclusivity regulations.

**Supplementary File 6. Opportunities for cash-in, cash-out (CICO) network and agent expansion.**

This file contains the results of supplemental targeted searches surrounding opportunities and enabling policies for CICO network expansion.
