# Supplementary material for: Mobile money and branchless banking regulations affecting cash-in, cash-out networks in low- and middle-income countries
Source: Gates Open Res. 2018 Nov 28;2:64. [Version 1] doi: 10.12688/gatesopenres.12876.1 (PMC6610045; doi:10.12688/gatesopenres.12876.1)
Supplement: Supplementary file 1 [file gatesopenres-2-13965-s0000.tgz › 9fc226f7-9c4e-42bb-a2a5-81bd6b69a549_Supplementary_File_1_Summary_of_Search_Strings_Used.docx]

**Supplementary File 1 – Summary of search strings used to compile literature on cash-in, cash-out (CICO) regulations.**

| **Search String** | **Google** | | | **Google Scholar** | | | **Scopus** | | |
| --- | --- | --- | --- | --- | --- | --- | --- | --- | --- |
|  | Results Returned | Documents Reviewed | New Relevant Results Identified | Results Returned | Documents Reviewed | New Relevant Results Identified | Results Returned | Documents Reviewed | New Relevant Results Identified |
| "cash in" OR "cash out" AND "digital finance" | - | - | - | 108 | 100 | 5 | 2 | 2 | 0 |
| "cash in" OR "cash out" AND "mobile banking" AND "regulation" | - | - | - | 1,440 | 100 | 3 | 9 | 9 | 0 |
| "cash*" AND "e-money" AND "regulation" | - | - | - | 3,430 | 80 | 2 | 52 | 40 | 0 |
| ("cash in" OR "cash out") AND ("digital finance" OR "digital financial") | - | - | - | 329 | 160 | 1 | 2 | 2 | 0 |
| ("cash in" AND "cash out") AND ("mobile banking" OR "mobile money" OR "e-money") AND "regulation" | - | - | - | 694 | 160 | 2 | 21 | 21 | 0 |
| site: [cgap.org](http://cgap.org/) "cash in" "cash out" regulat* | 222 | 170 | 1 | - | - | - | - | - | - |
| site: [gsma.com](http://gsma.com/) "cash in" ~"mobile banking" regulat* | 107 | 90 | 3 | - | - | - | - | - | - |
| "cash in" "cash out" "regulat*" ("Mobile banking" OR "digital finance" OR ~e-money) | 463 | 80 | 2 | - | - | - | - | - | - |
| ("cash in" OR "cash out") AND ("digital finance" OR "digital financial") AND regulat* | 403,300 | 100 | 5 | - | - | - | - | - | - |
| site: [helix-institute.com](http://helix-institute.com/) cash-in OR cash-out regulation | 43 | 41 | 0 | - | - | - | - | - | - |
| (Tanzania OR Nigeria OR Bangladesh OR Pakistan OR India OR Uganda OR Indonesia OR Kenya) And (“cash in” OR “cash out” OR “cash transaction”) AND (“mobile bank*” OR "digital finance") AND ~regulation | 49,500 | 100 | 2 | - | - | - | 7 | 7 | 0 |
| ATM AND (“cash in” OR “cash out”) AND regulation AND digital | 820,000 | 80 | 1 | - | - | - | 4 | 4 | 0 |
| ATM AND (“cash in” OR “cash out”) AND regulation AND (Tanzania OR Nigeria OR Bangladesh OR Pakistan OR India OR Uganda OR Indonesia OR Kenya) | 3,970 | 80 | 4 | - | - | - | 3 | 3 | 0 |
| branchless AND banking  AND  (~regulation OR ~regulatory) | - | - | - | - | - | - | 134 | 134 | 8 |
| ("cash in" OR "cash out") AND ("digital finance" OR "digital financial") AND "regulation" AND "impact" | 71,900 | 100 | 0 | 231 | 80 | 2 | - | - | - |
| ("cash in" OR "cash out") AND ("mobile banking" OR "mobile money" OR "e-money") AND "regulation" AND "impact" | 287,000 | 160 | 0 | 896 | 120 | 1 | - | - | - |
