# Supplementary material for: Mobile money and branchless banking regulations affecting cash-in, cash-out networks in low- and middle-income countries
Source: Gates Open Res. 2018 Nov 28;2:64. [Version 1] doi: 10.12688/gatesopenres.12876.1 (PMC6610045; doi:10.12688/gatesopenres.12876.1)
Supplement: Supplementary file 3 [file gatesopenres-2-13965-s0002.tgz › 18fb1bc4-0d0d-4c2d-a904-861e478ea4f6_Supplementary_File_3_Typology_of_CICO_Regulatory_Options.docx]

# Supplementary File 3 – Typology of cash-in, cash-out (CICO) regulatory decision options.

# This table outlines key decisions that countries can make to regulate CICO networks and highlights how each focus country has approached these decisions. Further detail on specific regulations is provided in Supplementary File 2: Coding spreadsheet of cash-in, cash-out (CICO) network regulations.

**Table S31. Typology of cash-in, cash-out (CICO) regulatory decision options.**

| **Regulatory Question** | **Regulatory Decision Options** | **Sub-Options** | **Countries Pursuing this Option** |
| --- | --- | --- | --- |
| What are the **KYC requirements** for mobile money? | 1a. Same KYC requirement as traditional banks | 1ai. For opening accounts | India; Indonesia; Nigeria; Pakistan; Tanzania |
|  |  | 1aii. For conducting transactions | Bangladesh; India; Kenya; Nigeria; Tanzania; Uganda |
|  | 1b. Less stringent KYC for mobile money accounts | 1bi. For opening accounts | India; Indonesia; Nigeria; Pakistan |
|  |  | 1bii. For conducting transactions | Bangladesh; India; Indonesia; Nigeria |
| Are there **fees** included with mobile money? | 2a. Fees originating from financial service provider (bank or non-bank) | 2ai. Fees are permitted | Bangladesh; India; Kenya; Nigeria; Pakistan; Tanzania; Uganda |
|  |  | 2aii. Fees are restricted | Nigeria |
|  | 2b. Additional fees originating from agents | 2bi. Fees are permitted | Nigeria |
|  |  | 2bii. Fees are restricted | Bangladesh; India; Kenya; Nigeria; Tanzania; Uganda |
|  | 2c. Restrictions on types of fees |  | Indonesia; Nigeria |
|  | 2d. Agent compensation (either from fees, commission, or some other revenue-sharing structure) |  | Bangladesh; India; Pakistan; Tanzania; Uganda |
| Are there (minimum or maximum) **caps** on mobile money? | 3a. Caps on balances held by customers |  | India; Indonesia; Nigeria; Pakistan; Tanzania |
|  | 3b. Caps on balances held  by agents |  | India; Kenya; Nigeria |
|  | 3c. Caps on transactions completed by customers |  | Bangladesh; India; Kenya; Nigeria; Pakistan; Uganda |
|  | 3d. Caps on transactions completed by agents |  | Bangladesh; Kenya; Uganda |
|  | 3e. Caps on other activities |  | India |
| Are there regulations about being **interoperable**? | 4a. Interoperability between multiple mobile money providers | 4ai. Is interoperability mandated? | Bangladesh; Indonesia; Kenya; Nigeria; Pakistan; Tanzania; Uganda |
|  |  | 4aii. Is interoperability encouraged or permitted? | Bangladesh; India |
|  | 4b. Interoperability between mobile money providers and banks | 4bi. Is interoperability mandated? | Bangladesh; Indonesia; Kenya; Nigeria; Pakistan; Tanzania; Uganda |
|  |  | 4bii. Is interoperability encouraged or permitted? | Bangladesh; India |
| Which entities are able or unable to use **agents**? | 5a. Banks' use of agents | 5ai. Can use agents | Bangladesh; India; Indonesia; Kenya; Nigeria; Pakistan; Tanzania; Uganda |
|  |  | 5aii. Restrictions on use of agents | Indonesia |
|  | 5b. MNOs/non-banks' use of agents | 5bi. Can use agents | Bangladesh; Indonesia; Kenya; Nigeria; Tanzania; Uganda |
|  |  | 5bii. Restrictions on use of agents | Nigeria |
| Are there **reporting** requirements? | 6a. Reporting requirements for agents | 6ai. Locations | Bangladesh; Indonesia; Kenya; Nigeria; Pakistan; Tanzania; Uganda |
|  |  | 6aii. Characteristics (e.g., gender, rural vs. urban) | Bangladesh; Kenya |
|  |  | 6aiii. Activities (e.g., transactions, account openings) | Bangladesh; India; Indonesia; Kenya; Nigeria; Tanzania; Uganda |
|  | 6b. Reporting requirements for other CICO networks | 6bi. Locations | Bangladesh; Nigeria |
| Are there requirements for who can and cannot be an agent? | 7a. Requirements on who can be an agent | 7ai. Requirements that must be met (e.g., business license) | Bangladesh; India; Indonesia; Kenya; Nigeria; Pakistan; Tanzania; Uganda |
|  |  | 7aii. Types of entities that can be agents (e.g. post offices, etc.) | Bangladesh; Kenya; Nigeria; Uganda |
|  |  | 7aiii. Dedicated agents (e.g., agents cannot have a side business like a bodega) | India |
|  | 7b. Requirements on who cannot be an agent |  | Bangladesh; India; Kenya; Nigeria; Tanzania; Uganda |
| Are there requirements for **agent exclusivity?** | 8a. Agent exclusivity is required | 8ai. Of all agents | Bangladesh; Indonesia |
|  |  | 8aii. Of some agents | Bangladesh; India |
|  | 8b. Agent exclusivity is permitted | 8bi. Of all agents | Pakistan |
|  |  | 8bii. Of some agents |  |
|  | 8c. Agent exclusivity is forbidden | 8ci. Of all agents | Kenya; Nigeria; Tanzania; Uganda |
|  |  | 8cii. Of some agents |  |
| Are there **functions or services** that agents must or cannot provide? | 9a. Functions/services that agents must provide | 9ai. Requirements on cash/e-float | Bangladesh; India; Kenya; Pakistan; Tanzania |
|  |  | 9aii. Other functions/services | Bangladesh; Nigeria |
|  | 9b. Functions/services that agents cannot provide |  | Bangladesh; India; Indonesia; Kenya; Nigeria; Pakistan; Tanzania; Uganda |
| Are there processes to **authenticate** agents? | 10a. Processes to authenticate agents by banks | 10ai. During registration | Bangladesh; India; Indonesia; Kenya; Nigeria; Pakistan; Tanzania; Uganda |
|  |  | 10aii. Ongoing | Bangladesh; India; Indonesia; Kenya; Nigeria; Pakistan; Tanzania; Uganda |
|  | 10b. Processes to authenticate agents by customers |  | Bangladesh; India; Kenya; Nigeria; Tanzania; Uganda |
| Are there distinctions made about **classes of agents**? | 11. Classes of agents |  | Bangladesh; India; Nigeria; Pakistan; Tanzania |
