# Supplementary material for: Mobile money and branchless banking regulations affecting cash-in, cash-out networks in low- and middle-income countries
Source: Gates Open Res. 2018 Nov 28;2:64. [Version 1] doi: 10.12688/gatesopenres.12876.1 (PMC6610045; doi:10.12688/gatesopenres.12876.1)
Supplement: Supplementary file 4 [file gatesopenres-2-13965-s0003.tgz › 040ba941-8092-4116-8250-6cb28af74e7a_Supplementary_File_4_CICO_Regulation_Summaries_by_Country.docx]

# Supplementary File 4 – Cash-in, cash-out (CICO) regulation summaries by country.

# These tables outline the key characteristics of CICO regulations by country. “Regulatory Decision Options” chosen by each country are noted in parentheses, following the typology provided in Supplementary File 3 (e.g., *regulatory decision option* 1ai = KYC requirements for opening accounts). Further detail on specific regulations is provided in Supplementary File 2: Coding spreadsheet of cash-in, cash-out (CICO) network regulations.

## Table S41. Summary of cash-in, cash-out (CICO) regulation in Bangladesh.

| **Type of regulations** | **Description** | **Regulation** |
| --- | --- | --- |
| Know Your Customer Requirements | Bangladesh's earliest KYC regulation (2011) requires a KYC Profile Form for all mobile accounts. This profile form includes information on the account holder's identity, as well as bank account information and purpose of the transaction, among others (*regulatory decision option* 1aii). A later regulation (2013) built upon this and required two-factor authentication for all mobile money accounts (*regulatory decision option* 1aii). However, a 2015 regulation stipulating that transactions must be authenticated using two-factor authentication states that mobile account utilized for low value transactions should be subject to risk-proportionate, simplified KYC procedures (*regulatory decision option* 1bii). Mobile accounts opened in the names of businesses, utilities, or other entities subject to the full KYC procedures that are applicable for regular bank accounts. Agents are not permitted to open mobile money accounts themselves. | Guidelines on Mobile Financial Services (MFS) for the Banks (2011), Guidelines on Agent Banking for the Banks (2013), Guidance Note for Approval and Operation of Agent Banking Activities of Banks (2013), Regulatory Guidelines for Mobile Financial Services (MFS) in Bangladesh (2015), Prudential Guidelines for Agent Banking Operation in Bangladesh (2017) |
| Interoperability | In a 2004 regulation, the Bangladesh Telecommunication Regulatory Commission encouraged interoperability enabled through platforms such as the National Payments Switch (NPS) or bKash, and stated that the commission may impose conditions to ensure this interoperability in the future (*regulatory decision option* 4aii). A 2011 regulation stated that banks may link their mobile financial services with those of other banks for the convenience of users (*regulatory decision option* 4bii). In 2015, regulation stated that inter-bank, or multi-bank interoperability is required for all MFS platforms (*regulatory decision option* 4bi). This interoperability was to be facilitated in part through developing linkages to platforms such as the National Payments Switch (NPS) (regulatory decision 4ai). Bangladesh's most recent regulation (2017) reiterated this, stipulating that banks must ensure that agent banking systems have interoperability functions (*regulatory decision option* 4bi). | Interconnection Regulations (2004), Guidelines on Mobile Financial Services (MFS) for the Banks (2011), Regulatory Guidelines for Mobile Financial Services (MFS) in Bangladesh (2015), Prudential Guidelines for Agent Banking Operation in Bangladesh (2017) |
| Institution types that can use agents (bank/non-bank) for financial transactions | Early regulation (2011) stipulated that Bangladesh's bank-led model of branchless banking may operate through appointed agents, facilitated by MNOs/Solution Providers (*regulatory decision option*5bi).  Later regulation (2013) reiterated that the agents of MNOs may conduct agent banking. Again in 2015, regulation stated that MPS platforms may enter into engagements with MNOs for agent banking.  A 2013 regulation stipulated that banks are permitted to conduct agent banking, which can include the agents of MNOs (*regulatory decision option* 5ai). An additional 2013 regulation required that banks to have at least two rural agent banking outlets to have one urban agent banking outlet, and stated that banks must prioritize rural areas for their operation of agent banking. This regulation also stipulated that "bank related persons" may not be agents or sub-agents. The most recent regulation (2017) reiterated that banks may provide banking services through agents. | Guidelines on Mobile Financial Services (MFS) for the Banks (2011), Guidance Note for Approval and Operation of Agent Banking Activities of Banks (2013), Guidelines on Agent Banking for the Banks (2013), Regulatory Guidelines for Mobile Financial Services (MFS) in Bangladesh (2015), Prudential Guidelines for Agent Banking Operation in Bangladesh (2017) |
| Use of exclusive agents | Two 2013 regulations stipulated that while agents may partner with more than one bank at a time, retail agents or sub agents may only represent and offer banking services of a single bank (*regulatory decision option* 8aii). The most recent regulation (2017) states that agents are not permitted to enter into agent banking contracts with more than one bank (*regulatory decision option* 8ai). | Guidelines on Agent Banking for the Banks (2013), Guidance Note for Approval and Operation of Agent Banking Activities of Banks (2013), Regulatory Guidelines for Mobile Financial Services (MFS) in Bangladesh (2015), Prudential Guidelines for Agent Banking Operation in Bangladesh (2017) |
| Agent selection (exclusion & pre-existing requirements) | Early regulation (2013) states that an agents must have sound financial capacity and strong, IT and electronic communication infrastructure, that is compatible with mobile money features (e.g., can integrate with Person Identification Number (PIN) pads, etc.). This regulation also stipulates that sub-agents must have physical infrastructure (e.g. at least one computer) and must have at least two full-time employees (*regulatory decision option* 7ai). An additional regulation from 2013 states that agents must have the competence to support mobile money activities, be financially sound, have a strong business reputation, and have the necessary infrastructure to support mobile money services, including technological, security, reporting and monitory capabilities (*regulatory decision option* 7ai). Later regulation (2015) reiterates these requirements for selecting agents by MFS platforms. Regulation from 2013 also identifies entities that can be engaged as agents. A variety of entities are eligible, and include NGOs, MFIs, post offices, pharmacies, offices of local government institutions, and agents of MNOs, among others (*regulatory decision option* 7aii). A 2015 regulation states that those entities with an extensive network of service delivery outlets (e.g., NGO MFIs, MNOs, government’s Postal Department etc.) are eligible to be wholesale/retail field level service delivery agents (*regulatory decision option* 7aii). The most recent regulation (2017) includes separate eligibility requirements for master agents and unit agents, however generally entities identified in 2017 regulation are still eligible and the regulation stipulates that any other entity which Bangladesh Bank may prescribe or authorize is eligible (*regulatory decision options* 7ai & 7aii). Both requirements for master and unit agents include requirements related to agent location, financial stability, and business licenses or permits (*regulatory decision option* 7ai). More stringent eligibility requirements exist for Master Agents, including that it must be an entity with multiple branches or outlets, it must employ at least two person with the required managerial and financial expertise, and it must carry out business activities on an ongoing basis, among others.   Two 2013 regulations indicate that an individual who has defaulted on a loan or who has been convicted under any criminal proceedings cannot be an agent (*regulatory decision option* 7b). This requirement is reiterated in a 2015 regulation. The most recent regulation (2017) indicates that bank officials or bank related persons are not eligible to be agents. Bank officials are not eligible to become agents with one year of their retirement or resignation. This regulation also states that defaulters, bankrupts, or persons convicted by a court of law up to three years after completion of sentences or penalties, or persons under investigation are not eligible to be agents. | Guidelines on Agent Banking for the Banks (2013), Regulatory Guidelines for Mobile Financial Services (MFS) in Bangladesh (2015), Prudential Guidelines for Agent Banking Operation in Bangladesh (2017) |
| Agent Compensation | Banks must pay a reasonable fee/commission to their agents. (*regulatory decision option* 2d) | Guidelines on Agent Banking for the Banks (2013) |
| Agent Services | A 2013 regulations states that agents must provide cash deposit and withdrawal services at a minimum (*regulatory decision option*9aii). Additionally, regulation from 2013 prohibits agents from giving final approval of opening of bank accounts and issuance of bank cards/cheques, dealing with loans and financial appraisal, encashing cheques or dealing in foreign currency (*regulatory decision option* 9b). The most regulation (2017) states that agents are not permitted to: open accounts, grant loans or carry out any appraisal function for purposes of opening an account or granting of a loan or any other facility; make debit or credit transactions using cheque; or transact in foreign currency (*regulatory decision option* 9b). | Guidelines on Agent Banking for the Banks (2013), Prudential Guidelines for Agent Banking Operation in Bangladesh (2017) |
| Agent e-float | A 2013 regulation requires that agents deposit a fixed amount of money or have a credit limit with the bank (*regulatory decision option* 9ai). An additional 2013 regulation restates this requirement. In addition to this, the regulation stipulates that banks will provide overdraft/credit facility to each agent, depending on the number of sub-agents under their authority (*regulatory decision option* 9ai). Credit should be no more than Tk. 100,000/- (One Lakh) per sub-agent. | Guidelines on Agent Banking for the Banks (2013), Prudential Guidelines for Agent Banking Operation in Bangladesh (2017) |
| Different Agent Classes | Early regulation (2013) identifies sub agents as entities who work under an agent and run the agent banking activities in a specific outlet at the customer end point (*regulatory decision option* 11). The most recent regulation (2017) indicates that classes of agents can include master agents and unit agents, and outlines the eligibility requirements for each. | Guidance Note for Approval and Operation of Agent Banking Activities of Banks (2013), Prudential Guidelines for Agent Banking Operation in Bangladesh (2017) |
| Caps & Fees | Early regulation (2011) states that banks may fix charges for mobile financial services, which will be under Bangladesh Bank oversight (*regulatory decision option* 2ai). Later regulation (2017) reiterates that banks are permitted to charge customers a fee or commission. A 2013 regulation states that agents can charge fees on behalf of banks, but are not permitted to charge customers fees or commissions directly (*regulatory decision option* 2bii). The most recent regulation (2017) reiterates this, stating that agents are required to collect fees, charges and commissions on behalf of banks, but may not charge customers directly.  Early regulation (2011) states that Bangladesh Bank will fix MFS transaction limits and overall caps (per customer/ per month) as and when needed (*regulatory decision option* 3c). Later regulation (2013) restricts clients to a withdrawal limit of Tk. 50,000.00 (Fifty Thousand) per day.  A 2015 regulation states that caps on transaction size will be proportionate to the account; low value accounts intended for low income individuals will have transaction size and frequency limits set by Bangladesh Bank. The most recent regulation (2017) sets a maximum number and volume of transaction by account type. Different limits exist for current accounts, savings accounts and special notice deposits. However, these limits may be exceeded if banks are given at least one day advance notice, or if approval is given by Managing Director/Chief Executive Officer of the bank. A 2013 regulation stipulates that sub-agents are restricted to a maximum of two transactions per client per day (*regulatory decision option* 3d). | Guidelines on Mobile Financial Services (MFS) for the Banks (2011), Guidelines on Agent Banking for the Banks (2013), Guidance Note for Approval and Operation of Agent Banking Activities of Banks (2013), Regulatory Guidelines for Mobile Financial Services (MFS) in Bangladesh (2015), Prudential Guidelines for Agent Banking Operation in Bangladesh (2017) |
| Agent Reporting Requirements | Early regulation (2011) required banks to report the names and addresses of all agents and cash points to the Department of Currency Management and Payment System (DCMPS), Bangladesh Bank on a monthly basis, and publish this list on its website (*regulatory decision option* 6ai & 6aiv). This regulation also required banks to report monthly on various transactions, including inward foreign remittances and other mobile financial transactions (*regulatory decision option* 6aiii). A 2013 regulation reiterated this requirement and stated that names and addresses of agents must also be submitted to the Green Banking and CSR Departments of Bangladesh Bank. This regulation also stipulated that required banks to submit an information sheet on proposed agents included just information as organizational history, financial strength and number of sub agents, among other characteristics (*regulatory decision option* 6aii). A 2015 regulation states that banks must provide a list of the names and locations of all retail agents and cash points when submitting a MFS platform application (*regulatory decision option* 6ai & 6aiv).  This regulation also required banks to submit monthly summaries on payment service transactions (*regulatory decision option* 6aiii). The most recent regulation (2017) states that banks are required to publish a list of all agents within its branches, at agent banking offices and on their website (*regulatory decision option* 6ai). This regulation also stipulates that banks must submit quarterly reports of agent activities, including value, volume and geographical distribution of transactions, and reporting on urban vs rural and male vs female, among other characteristics (*regulatory decision option* 6aii & 6aiii). | Guidelines on Mobile Financial Services (MFS) for the Banks (2011), Guidelines on Agent Banking for the Banks(2013), Guidance Note for Approval and Operation of Agent Banking Activities of Banks (2013), Regulatory Guidelines for Mobile Financial Services (MFS) in Bangladesh (2015), Prudential Guidelines for Agent Banking Operation in Bangladesh (2017) |
| Agent Authentication and Due Diligence | Early regulation (2011) required that banks have clear and well documented agent selection policies and procedures, and required banks to monitor activities of agents on a regular basis (*regulatory decision option* 10ai & 10aii). A 2013 regulated required banks to submit agent and sub agent due diligence policies and procedures as part of their agent banking application (*regulatory decision option* 10ai). Additionally, 2013 regulation required banks to formulate internal audit policies for monitoring and controlling agents, including visiting agent locations at regular intervals to ensure agent compliance with rules and regulations (*regulatory decision option* 10aii). Later regulation (2015) stated that MFS platforms should have internal controls and audit processes including procedures for routine oversight of agents and customer satisfaction levels (*regulatory decision option* 10aii). The most recent regulation (2017) states that banks must conduct due diligence prior to engaging an agent, which must include at a minimum checks of agents at specified intervals, as well as procedures for proactive agent management including identifying warning signals and associated corrective action (*regulatory decision option* 10ai & 10aii).   A 2013 regulation required agents to clearly display the name, logo, contact address and telephone number of the bank so that customers know the agent is providing services on behalf of the bank (*regulatory decision option* 10b). Additionally, this regulation required banks to make agents, their activities and limitations known to the public. The most recent regulation (2017) requires agents to clearly display and disclose to customers their business license, bank approval letter and list of eligible services that they may provide (*regulatory decision option* 10b). | Guidelines on Mobile Financial Services (MFS) for the Banks (2011), Guidelines on Agent Banking for the Banks(2013), Guidance Note for Approval and Operation of Agent Banking Activities of Banks (2013), Regulatory Guidelines for Mobile Financial Services (MFS) in Bangladesh (2015), Prudential Guidelines for Agent Banking Operation in Bangladesh (2017) |
| Other: Agent Banking Priorities | In establishing agent banking outlets or banking service outlets banks are required to give priority to areas where formal financial services are unavailable, including areas where there is no bank branch within a 1 km radius, rural areas, chars, islands and other geographical areas with limited accessibility. Agent banking outlets should be distributed equally among these previously unreached areas. At a minimum banks should maintain a ratio of 3:1 for rural and urban agent banking outlets.   Banks are encouraged to promote small businessmen and women entrepreneurs for unit agents, and should employ women officials for facilitating acquisition of rural women clients. Additionally, banks should enact policies which prioritize low-income households, cottage, micro and small businesses without access to formal banking services. | Prudential Guidelines for Agent Banking Operation in Bangladesh (2017) |

## Table S42. Summary of cash-in, cash-out (CICO) regulation in India.

| **Type of regulations** | **Description** | **Regulation** |
| --- | --- | --- |
| Know Your Customer Requirements | An initial regulation in 2010 stated that Business Correspondents should comply with previously outlined KYC procedures for opening accounts (*regulatory decision option*1ai). In 2014, a Master Circular reinforced this language, despite 2012 language that suggested modified KYC requirements may be developed. A 2016 regulation added that stated that previously outlined KYC guidelines are applicable to mobile-based banking services, and that the dispersal of funds as an agent is only permitted after identification (two-factor authentication, including mPIN) (*regulatory decision option* 1aii). However in 2012, regulations suggested that a simplified KYC requirements be developed for mobile-linked "No Frills" accounts, but did not provide these simplified requirements until 2014 (*regulatory decision option* 1bi). In 2014, the Reserve Bank of India released simplified KYC requirements for opening a bank account, and created less stringent requirements for low/medium risk customers compared to high risk customers (*regulatory decision option* 1bi) and additional guidelines were released in 2014 that these simplified KYC requirements for opening accounts also apply to "small account" transactions (*regulatory decision option* 1bii). | Financial Inclusion by Extension of Banking Services – Use of Business Correspondents (BCs) (2010); The Mobile Banking (Quality of Service) Regulations (2012); Master Circular on Branch Authorization (2014); RBI’s Recent simplified KYC Measures For Public Awareness (2014); Guidelines for Licensing of “Payments Banks” (2014); Master Circular – Mobile Banking transactions in India – Operative Guidelines for Banks (2016); Master Direction - Know Your Customer (KYC) Direction (2016) |
| Interoperability | In 2012, a 2010 regulation on financial inclusion by extension of banking services was amended to allow for interoperability; this amendment states that agents and sub-agents can be interoperable among one another as long as basic requirements, such as operating on the Core Banking Solution platform, are met (*regulatory decision option* 4aii & 4bii). Another regulation in 2012 proposed a system for sharing interoperable central payments across Banking Correspondents, banks or associated financial institutions, and mobile service providers, a 2014 regulation reinforced the 2010 regulation/2012 amendment, permitting agents and sub-agents to be interoperable (*regulatory decision option*4aii & 4bii). In 2016, a Master Circular stated that interoperability between banks, irrespective of the mobile network, is a long-term goal for all of India (*regulatory decision option* 4bii). Additionally, two regulations in 2016 allowed for interoperability among Business Correspondents, except for the opening of savings, current, and deposit accounts (*regulatory decision option* 4bii). | Financial Inclusion by Extension of Banking Services – Use of Business Correspondents (BCs) (2010); The Mobile Banking (Quality of Service) Regulations (2012); Master Circular on Branch Authorization (2014); Guidelines for Licensing of “Payments Banks” (2014); Master Circular – Mobile Banking transactions in India – Operative Guidelines for Banks (2016); Operating Guidelines for Payments Banks (2016); Operating Guidelines for Small Finance Banks (2016) |
| Institution types that can use agents (bank/non-bank) for financial transactions | A 2010 regulation noted that commercial banks may engage Business Correspondents.  2012 regulation was amended in 2013 to stipulate that banks can use agents to provide banking services through mobile phones. In 2014, a Master Circular added to the 2010 regulation regarding commercial banks and Business Correspondents, and added that banks are permitted to use the services of intermediaries in providing financial and banking services through the Business Correspondent model. Another Master Circular issued the same year (2014) specified the Regional Rural Banks may use intermediaries as well.  A 2016 regulation reinforced that banks may use Business Correspondents. And finally in 2016, two sets of guidelines--one for Payment Banks and one for Small Finance Banks--allowed these entities to engage "access points," or intermediaries to provide their financial services (*regulatory decision option* 5ai). | Financial Inclusion by Extension of Banking Services – Use of Business Correspondents (BCs) (2010); The Mobile Banking (Quality of Service) Regulations (2012); Master Circular on Branch Authorization (2014); Master Circular on Branch Licensing - Regional Rural Banks (RRBs) (2014); Master Circular – Mobile Banking transactions in India – Operative Guidelines for Banks (2016); Operating Guidelines for Payments Banks (2016); Operating Guidelines for Small Finance Banks (2016) |
| Use of exclusive agents | A 2010 regulation stated that Business Correspondents may partner with multiple banks, however a retail or sub-agent may only partner with one bank. A regulation 2014 reinforced the 2010 regulation, and also stipulated that BCs much maintain separate databases for each partner bank (*regulatory decision option* 8aii). | Financial Inclusion by Extension of Banking Services – Use of Business Correspondents (BCs) (2010); Master Circular on Branch Authorization (2014) |
| Agent selection (exclusion & pre-existing requirements) | A regulation in 2010 specified which different types of entities may engage as a Business Correspondent. This regulation also required BCs to be attached to and under the oversight of a specific bank branch. It specifies that a retail outlet/sub-agent of a BC must be no further than 50 km from the bank in rural, semi-urban, and urban areas; and may not exceed a distance of 5 km in metropolitan centers. This 2010 regulation was updated in 2014 to allow banks to engage non-deposit taking institutions as BCs, if requirements are met. The distance requirement from 2010 was removed. A separate 2014 regulation also provides a list of entities that may serve as an intermediary or Business Correspondent for banks.  A set of 2014 guidelines state that preference will be given to Payment Banks that provide access points in under-banked locations in the Northeast, East, and Central regions of the country. It also states that Payment Banks should ensure that their reach remote areas and that at least 25 percent of access points should be in rural centers.  A 2016 regulation exempted Payment Banks from a requirement to have a base branch for a certain number of access points or Business Correspondents, and a separate 2016 regulation states that only Business Correspondents which conduct online transactions or use PoS terminals for transactions will be permitted (*regulatory decision option* 7ai).  Additionally, a 2014 regulation prohibits Payment Banks from setting up subsidiaries that undertake non-banking financial services (*regulatory decision option* 7b). | Financial Inclusion by Extension of Banking Services – Use of Business Correspondents (BCs) (2010); Master Circular on Branch Authorization (2014); Guidelines for Licensing of “Payments Banks” (2014); Operating Guidelines for Payments Banks (2016); Operating Guidelines for Small Finance Banks (2016) |
| Agent Compensation | A 2010 regulation states that banks can pay a reasonable commission/fee to their Business Correspondents, the rate and amount of which may be reviewed periodically. The commission structure or incentive mechanism should be devised in a manner that an increase in only the number of clients served or the transaction volume does not drive the commission. This was reinforced in a 2016 regulation (*regulatory decision option* 2d). | Financial Inclusion by Extension of Banking Services – Use of Business Correspondents (BCs) (2010); Master Circular on Branch Authorization (2014) |
| Agent Services | A 2014 regulation prohibits Payment Banks from undertaking lending activities, accepting NRI deposits, issuing credit cards, or setting up subsidiaries to undertake non-banking financial services (*regulatory decision options* 9b & 7aiii). This same regulation also requires that Payment Banks ensure that non-financial service activities be kept separate from banking and financial services; it also requires that "Payment Bank" be clearly stated in the title, and that these banks invest 75 cents of demand deposit balances in Government Securities or Treasury Bills, have a minimum equity capital of Rs. 100 crore, and a minimum capital adequacy ratio (ratio depends on tier) (*regulatory decision option* 9ai). In 2016, a set of guidelines stipulate that when a Payment Bank acts as a Business Correspondent for a bank, the Payment Bank's own BCs may not open deposits or review KYC documentation for the bank (*regulatory decision option* 9b). | Operating Guidelines for Payments Banks (2016) |
| Caps & Fees | A 2010 regulation requires Business Correspondents to specify suitable limits on individual customer payments, but no value was specified; this was reinforced in a 2014 regulation (*regulatory decision option* 3a). This 2010 regulation also stated that: banks are permitted to collect reasonable service charges from customers (*regulatory decision option*2ai); Business Correspondents may not charge any fee to customers directly for services rendered on behalf of the bank, which was reinforced in a 2014 regulation (*regulatory decision option* 2bii); and that Business Correspondents should specify suitable limits on cash holding by intermediaries, which was also reinforced in a 2014 regulation (*regulatory decision option* 2bii). The 2014 regulation also added guidelines stating that Payment Banks may not provide balances that exceed Rs. 100,000 per customers until the Reserve Bank of India has assessed the individual PB's performance. Meanwhile, small account balances may not exceed Rs. 50,000 (*regulatory decision option* 3a) and that Payment Banks may not allow transfers for small accounts that exceed Rs. 10,000 per month (*regulatory decision option* 3c). In 2016, a Master Circular stated that the maximum cash out value for transfers shall be Rs. 10,000 per transaction; banks may place additional caps on the number of transaction, subject to a maximum value of Rs. 25,000 per month; and regarding mobile banking, banks may offer services with no maximum caps for daily transactions, depending on the bank's own risk perception (*regulatory decision option* 3c). In 2014, guidelines stated that Payment Banks may not allow credits that exceed Rs. 1 lakh for small accounts (*regulatory decision option* 3e). | Financial Inclusion by Extension of Banking Services – Use of Business Correspondents (BCs) (2010); Master Circular on Branch Authorization (2014); Guidelines for Licensing of “Payments Banks” (2014); Master Circular – Mobile Banking transactions in India – Operative Guidelines for Banks (2016) |
| Agent Reporting Requirements | A 2010 regulation stated that banks may place information on their Business Correspondents on their websites, and that annual reports should include progress towards extending financial services through BC networks (*regulatory decision option* 6aiii). | Financial Inclusion by Extension of Banking Services – Use of Business Correspondents (BCs) (2010) |
| Agent Authentication and Due Diligence | A 2010 regulation states that: banks must carry out due diligence reviews before engaging potential Business Correspondents, and provides specific items to review (*regulatory decision option* 10ai); banks should regularly monitor the activities of their BCs and conduct a detailed review of BC performance at least once per year (*regulatory decision option* 10aii); and agents and sub-agents may be required to post a sign indicating their status as a service provider to banks for customers (*regulatory decision option*10b). All three of these were reinforced in a 2014 regulation. Additionally, a 2016 regulation states that banks may carry out due diligence of individuals before appointing them as agents that conduct fund transfer services (*regulatory decision option* 10ai). | Financial Inclusion by Extension of Banking Services – Use of Business Correspondents (BCs) (2010); Master Circular on Branch Authorization (2014); Master Circular – Mobile Banking transactions in India – Operative Guidelines for Banks (2016) |
| Different Agent Classes | 2010 regulations state that while a BC can be a BC for more than one bank, at the point of customer interface, a retail outlet or sub-agent of a BC shall represent and provide banking services of only one bank (*regulatory decision option*11). 2012 regulations affirm the use of sub-agents. | Financial Inclusion by Extension of Banking Services – Use of Business Correspondents (BCs) (2010, 2012) |
| Agent e-float | No relevant regulations were found on this topic. |  |

## Table S43. Summary of cash-in, cash-out (CICO) regulation in Indonesia.

| **Type of regulations** | **Description** | **Regulation** |
| --- | --- | --- |
| Know Your Customer Requirements | Indonesia's earliest regulation pertaining to DFS (2003) created KYC requirements consistent with those of traditional banks for account opening (*regulatory decision option* 1ai). This regulation and subsequent required customers to submit their full name, identity document number, residential address, place and date of birth, nationality, phone number, occupation, sex, and biometric data or signatures when opening an account. Identity documents could include an identity card (KTP), driver's license (SIM), passport, or other official documents issued by Governments Agencies.  In 2014, new branchless banking regulation allowed for some simplified KYC requirements for certain accounts (*regulatory decision option* 1bi). For example, basic savings accounts could be opened with a reference letter from a local community leader. The most recent regulation (2017) further relaxes KYC requirements (*regulatory decision options* 1bi & 1bii). It applies simplified KYC requirements (both for opening accounts and conducting transactions) to be applied more broadly to those considered low risk. Low risk users could be based on the country or geographic area, product or service, or delivery channel. Low-risk products or services could include products or services made to support financial inclusion, enhancement of community welfare, poverty alleviation and/or aimed at persons with disabilities, or other products or services designed for a limited purpose, usability, or with limited features. Service providers are allowed to collect KYC documents via third parties (i.e., agents). | Regulation 3/10/PBI/2001, as amended by Regulations 3/23/PBI/2001 and 5/21/PBI/2003 (2003); Money Transfer Circular, BI Regulation 8/28/PBI/2006 (2006); Bank Indonesia Regulation Number: 11/12/PBI/2009 Concerning Electronic Money (2009); Circular No. 11/11/DASP Concerning Electronic Money (2009); No. 19 / POJK.03 / 2014: Branchless banking "laku pandai" (2014); Bank Indonesia Regulation Number: 19/10/PBI/2017 Concerning Implementation of Anti-Money Laundering (2017) |
| Interoperability | All e-money systems are required to be interoperable (*regulatory decision options* 4ai & 4bi). However, this is not the case for point-of-sale (POS) terminals, where commercial banks use non-interoperable proprietary systems. | Bank Indonesia Regulation Number: 11/12/PBI/2009 Concerning Electronic Money (2009); Circular No. 11/11/DASP Concerning Electronic Money (2009) |
| Institution types that can use agents (bank/non-bank) for financial transactions | Early regulation (2006) prohibited rural banks from providing financial services through agents, and prohibited money transferors from using agents (*regulatory decision option*5aii). However, regulation allowed for banks to use a tiered system of bank branches which included such mobile cash services as cash automobiles and cash boats. In 2009 regulations were revised to allow e-money issuers to partner with agents for e-money replenishment and cash withdrawal (though not in the context of money transfer) (*regulatory decision option* 5bi). Additionally, regulation in 2009 allowed banks to cooperate with payment points, where customers process bill payments and payment of salaries. In 2014 Indonesia introduced the pilot program Laku Pandai, which allowed banks meeting certain criteria in specific areas that were already providing mobile money services to partner with agents (*regulatory decision option* 5ai). This applied to banks that are Indonesian legal entities, have a risk profile grading of 1-3, have office networks in the eastern part of Indonesia and/or East Nusa Tenggara, and already provide mobile banking services. The most recent regulation (2017) allows banks to partner with agents to make a direct connection with a potential customer, including agents, cash payment points (TPT), and digital financial services (LKD) agents (*regulatory decision option*5ai). | Money Transfer Circular BI Regulation 8/26/PBI/2006 (2006); Circular No. 11/11/DASP Concerning Electronic Money (2009); Commercial Banks Regulation 1 1/1/PBI/2009 (2009); No. 19 / POJK.03 / 2014: Branchless banking "laku pandai" (2014); Bank Indonesia Regulation Number: 19/10/PBI/2017 Concerning Implementation of Anti-Money Laundering (2017) |
| Use of exclusive agents | The Laku Pandai pilot program in 2014 included a requirement for agent exclusivity (*regulatory decision option*8ai). | No. 19 / POJK.03 / 2014: Branchless banking "laku pandai" (2014) |
| Agent selection (exclusion & pre-existing requirements) | The earliest regulation concerning agent selection (2006) stipulated that agents must obtain a license as business activity administrator of money remittance in order to provide cash-in or cash-out services (*regulatory decision option* 7ai). With the introduction of the Laku Pandai pilot program in 2014, agents were required to be domiciled in the location where the program is conducted, and have had a viable source of income for the last 2 years (*regulatory decision option* 7ai).  Additionally, agents must have IT infrastructure and retail outlets that are sufficient to support the Laku Pandai business. | Money Transfer Circular; BI Regulation 8/28/PBI/2006 (2006); Circular No. 11/11/DASP Concerning Electronic Money (2009); No. 19 / POJK.03 / 2014: Branchless banking "laku pandai" (2014) |
| Agent Services | It is recommended that banks consider limiting the scope of services that agents can provide (*regulatory decision option*9b). However, it is ultimately up to the banks to determine the scope of services offered. | No. 19 / POJK.03 / 2014: Branchless banking "laku pandai" (2014) |
| Caps & Fees | Early regulation (2009) specified different caps on account balances and transactions for registered and unregistered mobile money users; caps on balances and transactions are lower for unregistered mobile money users (*regulatory decision option* 3a).  The maximum account balance for basic savings accounts is RP 20,000,000; there is no minimum account balance. The cap on cash-out transactions for basic savings accounts is Rp 5,000,000/month or Rp 60,000,000/year; there are no cash-in or cash-out minimums. A later regulation (2014) reiterated these limits (*regulatory decision option* 3a). In later rounds of regulations (2014) Indonesia stipulated that customers may only be charged for withdrawals and transfer-out services, and that all other transactions are free (*regulatory decision option* 2c).  No information is included on caps related to agents specifically. | Circular No. 11/11/DASP Concerning Electronic Money (2009), No. 19 / POJK.03 / 2014: Branchless banking "laku pandai" (2014) |
| Agent Reporting Requirements | Early regulation (2009) stipulated that information on cash service activities, including from mobile cash services and payment points, must be included in Bank financial reports (*regulatory decision option* 6aiii). Later regulation (2017) expanded these reporting requirements to include records related to all customer financial transaction, and information on agents (*regulatory decision options* 6ai & 6aiii). Service providers are required to report this information to Bank of Indonesia upon request. | Commercial Banks Regulation, Regulation 1 1/1/PBI/2009 (2009); Bank Indonesia Regulation Number: 19/10/PBI/2017 Concerning Implementation of Anti-Money Laundering (2017) |
| Agent Authentication and Due Diligence | Early regulation (2009) allowed for the collection of information on agents who conducted damaging actions and the creation of an agent "black list" to be shared among service providers (*regulatory decision option* 10aii). The most recent regulation (2017) allows Bank Indonesia to periodically monitor and evaluate agents to ensure compliance with regulations and procedures (*regulatory decision option* 10aii).   In 2014, regulation stipulated that agents must pass a banks' due diligence procedures (*regulatory decision option* 10ai). | Bank Indonesia No. 11/12/PBI/2009 Electronic money (2009), No. 19 / POJK.03 / 2014: Branchless banking "laku pandai" (2014); Bank Indonesia Regulation Number: 19/10/PBI/2017 Concerning Implementation of Anti-Money Laundering (2017) |
| Different Agent Classes | No relevant regulations were found on this topic. |  |
| Agent e-float | No relevant regulations were found on this topic. |  |
| Agent Compensation | No relevant regulations were found on this topic. |  |

## Table S44. Summary of cash-in, cash-out (CICO) regulation in Kenya.

| **Type of regulations** | **Description** | **Regulation** |
| --- | --- | --- |
| Know Your Customer Requirements | Early regulations (2010) required customers be identified using two factor authentication, at a minimum, when conducting transactions. Authentication methods include IDs, PINs, passwords, ATM card, secret code, or secret message among others (*regulatory decision option* 1aii). This requirement is restated in 2013 regulations. 2014 regulations required payment service providers to maintain a register of customers and their outstanding funds, which should include either the ID number or passport number of the customer (*regulatory decision option* 1aii). | Guidelines on Agent Banking (2010); Prudential Guidelines for Institutions Licensed Under the Banking Act (2013); The National Payment System Regulations, 2014 (2014) |
| Interoperability | In 2013, regulations required payment service providers to use systems capable of interoperability with other payments systems, both domestically and internationally. Additionally, payment service providers were encouraged to enter into interoperability agreements (*regulatory decision options* 4ai & 4bi). The most recent regulation (2014) reaffirms this. | The National Payment System Regulations, 2013 (2013); The National Payment System Regulations, 2014 (2014) |
| Institution types that can use agents (bank/non-bank) for financial transactions | Early regulations (2010) stated that Institutions as defined by the Banking Act, which include banks, mortgage finance companies, or a financial institutions, and the Microfinance Act, which include deposit-taking microfinance businesses, may use agents for financial services (*regulatory decision options* 5ai & 5bi). Additionally, any other body the Minister and the Central Bank allow may use agents for financial services. More recent regulation (2013) reaffirms this. | Guidelines on Agent Banking (2010); Prudential Guidelines for Institutions Licensed Under the Banking Act (2013) |
| Use of exclusive agents | Early regulations (2010) stated that institutions are not permitted to have exclusive contracts with agents (*regulatory decision option* 8ci). Agents must have separate contracts with each institution, and must have the capacity to contract with multiple institutions prior to doing so. 2013 and 2014 regulations reaffirm this (*regulatory decision option* 8ci). | Guidelines on Agent Banking (2010); The National Payment System Regulations, 2013 (2013); Prudential Guidelines for Institutions Licensed Under the Banking Act (2013); The National Payment System Regulations, 2014 (2014) |
| Agent selection (exclusion & pre-existing requirements) | The earliest regulation (2010) stipulated that prior to appointing an agent, an institution must show that an agent has a well-established commercial activity, and business license or permit, that has been active for at least 18 months, and has the capacity to provide banking services safely and efficiently (*regulatory decision option* 7ai). 2010 regulations stated that organizations eligible to become an agent include limited liability companies, sole proprietorships, partnerships, societies, cooperative societies, state corporations, public entities, and any other organization identified by the Central Bank (*regulatory decision option* 7aii). 2013 regulations required agents and partnering organizations to possess registrations, business licenses, or permits for a commercial activity (*regulatory decision option* 7ai). More specifically, the regulations stipulated that organizations must submit to the institution it plans to partner with a certificate of incorporation or registration of its business name, if applicable, a description of the commercial activity it has been carrying out for the last twelve months and the associated business license or permit, if applicable, bank statements for the last two years, if applicable, the agents physical location including postal address, GPS coordinates and telephone number, and evidence of an availability of funds (*regulatory decision option* 7ai). 2013 regulations also expanded the types of activities agents can be involved in to include either commercial or non-commercial activities and offered more specification on how an agent can prove their capacity, specifically that an agent must hold a payment account with a financial institution and show that they are financially sound (*regulatory decision option* 7ai). The most recent regulation (2014) reaffirms this. | Guidelines on Agent Banking (2010); E-Money Regulation (2013); The National Payment System Regulations, 2013 (2013); Prudential Guidelines for Institutions Licensed Under the Banking Act (2013); The National Payment System Regulations, 2014 (2014) |
| Agent Services | 2010 regulation stipulated that agents should show that they have funds available to cover all operations, including customer deposits and withdrawals when applying to become an agent (*regulatory decision option* 9ai). 2010 regulations also state that agents are not permitted to offer banking services on its own accord, offer any guarantee in favor of any institution or customer, open accounts, grant loans, undertake cheque deposit and encashment of cheques, transact foreign currency, or provide cash advances (*regulatory decision option* 9b). 2013 regulation stated that e-money is not a deposit within the meaning of the Banking Act, the Microfinance Act, or the SACCO Societies Act and is not subject to any deposit protection (*regulatory decision option* 9ai). Agent banking businesses are not permitted to be run by an employee or an associate of a bank. Agents shouldn't continue offering banking services if their initial commercial activity has ceases or significantly diminished, or if the agent has a proven criminal record involving fraud, dishonesty, integrity or any other financial impropriety. | Guidelines on Agent Banking (2010); Prudential Guidelines for Institutions Licensed Under the Banking Act (2013); The National Payment System Regulations, 2013 (2013) |
| Agent e-float | 2010 regulation stipulated that agents should show that they have funds available to cover all operations, including customer deposits and withdrawals when applying to become an agent (*regulatory decision option* 9ai). Limits on cash holdings by the agent should be included in every agent contract. 2013 regulation stated that e-money is not a deposit as defined by the Banking Act, the Microfinance Act, or the SACCO Societies Act and is not subject to deposit protection (*regulatory decision option* 9ai). | Guidelines on Agent Banking (2010) |
| Caps & Fees | 2010 regulations specified that limits on cash holdings by the agent should be included in every agent contract (*regulatory decision option* 3b). These regulations also stated that institutions should set prudent transaction limits for each type of transaction that take into account the risks associated with the agents locality (*regulatory decision option* 3d). The most recent legislation (2014) further specifies that individual transactions may not exceed 70,000 shillings and aggregate monthly transactions may not exceed 1 million shillings, however the bank may approve higher limits for specific e-money issuers (*regulatory decision option* 3c). Early regulations (2010) mandated that agents not be permitted to charge customers fees, and all contracts between an institution and an agent should stipulate this (*regulatory decision option* 2bii). In 2013 regulations stated that the fees applicable to the redemption of e-money and fees for balance inquiries should be made known to the customers upon account opening (*regulatory decision option* 2ai). | Guidelines on Agent Banking (2010); E-Money Regulations (2013); The National Payment System Regulations, 2014 (2014) |
| Agent Authentication and Due Diligence | 2010 regulations stated Agent Due Diligence procedures should be in place that at a minimum contain methods for identifying agents, initial due diligence procedures, regular due diligence checks and their specified intervals, and a list of warning signals and corrective actions (*regulatory decision options* 10ai & 10aii). 2010 regulation also state that institutions should ensure that a channel of communication exists through which customers may verify the authenticity of an agent, its location and the validity of the agents banking business. If requested by a customer, an agent should show a copy of their bank approval letter, appointment letter from the institution, and the license for the commercial activity the agent is undertaking (*regulatory decision option* 10b). These rules are reaffirmed in 2013 and 2014 regulations. 2013 regulations stated that in selecting agents an institution should exercise due diligence and conduct a suitability assessment of the proposed agents (*regulatory decision option* 10ai). 2013 regulation stated that the Bank is permitted to conduct on-site inspections of agents, and inspect the accounts and other documents of agents at any time to ensure compliance with the Laws of Kenya (*regulatory decision option* 10aii). Additionally, 2013 regulations specified that agents must clearly display the name of the institutions for which they are working, a list of banking services offered, a notice that services are provided subject to the availability of funds, a list of applicable fees and charges payable to the institution, the dedicated phone number customers can use to contact the institution, and the name, telephone number and location of the institutions branch that the agent reports to (*regulatory decision option*10b). | Guidelines on Agent Banking (2010); The National Payment System Regulations, 2013 (2013); Prudential Guidelines for Institutions Licensed Under the Banking Act (2013); The National Payment System Regulations, 2014 (2014) |
| Agent Reporting Requirements | Early regulations (2010) stated that every month, institutions must submit information to the Central Bank with details of the nature, value, volume, and geographical distribution of operations and transactions (*regulatory decision options* 6ai & 6aiii). 2013 regulations require e-money issuers to report to the bank monthly on the physical address, GPS coordinate, postal address, physical address and telephone number of each of its agents. Regulation from this year also stated that the application for agent banking (with agents names, physical locations, GPS co-ordinates, postal addresses, and telephone numbers, as well as the total population and economically active population for the areas in which they will operate) should be updated and submitted on a yearly basis with the agent banking renewal application (*regulatory decision options* 6ai & 6aii). The most recent regulation (2014) reaffirms that payment service providers should maintain records containing agents' names, physical addresses, postal addresses, and telephone numbers (*regulatory decision option* 6ai). | Guidelines on Agent Banking (2010); E-Money Regulation (2013); The National Payment System Regulations, 2013 (2013); Prudential Guidelines for Institutions Licensed Under the Banking Act (2013); The National Payment System Regulations, 2014 (2014) |
| Different Agent Classes | No relevant regulations were found on this topic. |  |
| Agent Compensation | No relevant regulations were found on this topic. |  |
| Other: Deposit Related Specifications | E-money is not a deposit within the meaning of the Banking Act, the Microfinance Act, or the SACCO Societies Act and is not subject to any deposit protection. | E-Money Regulations (2013) |

**Table S45. Summary of cash-in, cash-out (CICO) regulation in Nigeria.**

| **Type of regulations** | **Description** | **Regulation** |
| --- | --- | --- |
| Know Your Customer Requirements | Most regulations require the same KYC requirements as traditional banks: A 2015 regulation allows customers to be identified through a valid international passport, National Identity card, permanent voter's card, or driver's license when opening an account (*regulatory decision option* 1ai).   For transactions, early regulation (2007) required POS scanners to be updated to include Biometric Authentication in the form of fingerprint reader/scanners. It also required cardholders to present authentication to a merchant when requested. A 2013 regulation allows customers to be identified with an ID, PIN, password, payment card, secret code, or secret message when conducting transactions. This was reiterated in 2015. Further 2015 regulation stipulates that PINs shall be encrypted at point of entry on a mobile money user interface. It also states that pull-based transactions (credits through a mobile money solution) must be authorized by the account holder via a verifiable mode before the transaction is consummated. A 2016 regulation states that customers are required to show a document confirming their identity if requested by a merchant. POS are required to utilize Payment Card Industry PIN Transaction Security (PCI PTS) in accordance with the PCI Point-to-Point Encryption (P2PE) Solution Requirements (*regulatory decision option* 1aii).   Other regulations allow less stringent KYC for mobile money accounts: A 2013 regulations distinguishes between KYC requirements for three levels of accounts. Customers are required to provide basic information, such as a passport photo, name, place, and date of birth, gender, address, telephone number, etc. when opening low-value accounts (level 1) and medium-value accounts (level 2), and are required to comply with all KYC requirements when opening high-value accounts. Later regulation (2017) states that KYC Level 1 accounts are not required to provide Bank Verification Numbers when opening an account as part of the KYC documentation, though it is mandatory for Level 2 and 3 accounts (*regulatory decision option* 1bi).    For transactions, a 2014 regulation distinguishes between four types of online funds transfers, from low security to highly secured transfers, with varying required controls for each level (*regulatory decision option* 1bii). | Guidelines on Point-of-Sale (POS) Card Acceptance Services (2007); Circular on Introduction of Three-tiered Know Your Customer Requirements (2013); Guidelines for the Regulation of Agent Banking and Agent Banking Relationships in Nigeria (2013); Circular on Exposure Draft on the Framework for Licensing Super Agents (2014); Regulatory Framework for Mobile Money Services in Nigeria (2015); Guidelines on Mobile Money Services in Nigeria (2015); Guidelines on International Mobile Money Remittance Service in Nigeria (2015); Guidelines on Operations of Electronic Payment Channels in Nigeria (2016); Review of Daily MM Wallet Transaction & BVN Requirement for Mobile Money Wallet Holders (2017) |
| Caps & Fees | Four regulations relate to fees charged by the financial services provider: (1) a 2014 regulation creates a minimum fee for cash-in transactions (N35) and for cash-out transactions (N50) (*regulatory decision option* 2aii); (2) a 2014 regulation states that fees for remote-on-us ATM cash withdrawal are N65 per transaction, though the first three transactions in a given month are free (*regulatory decision option* 2c); (3) a 2015 regulation states that no airtime deductions shall be made in respect of charges on any transaction (*regulatory decision option* 2c), and (4) a 2016 regulation stipulates that fees for transactions must be agreed upon by service providers and financial institutions (*regulatory decision option* 2ai).    Early regulation (2007) outlined the distribution of fees for transactions and prohibited agents from charging an additional fee to customers for using their cards. A 2013 regulation states that agents cannot charge fees directly and that they are required to post all fees payable to the financial institution. (A 2014 regulation reiterates that agents are not permitted to charge customers fees directly.) (*regulatory decision option* 2bi) Recent regulation (2016) stipulates that fees for transactions must be agreed upon by service providers and financial institutions. Merchant limits should be based on the volume of business and type of commercial activities performed (*regulatory decision option*2bi). The same regulation prohibits merchants from charging customers who use a card a different price (*regulatory decision option* 2bii).    A 2013 regulation created distinct caps on balances and transactions for Low-Value Accounts (Level 1), Medium-Value (Level 2), and High-Value (Level 3) accounts. There is no minimum balance requirement when opening accounts for any of the levels. Another 2013 regulation states that limits on customer transactions and withdrawals are required to be included in contracts between agents and financial institutions.  A 2014 regulation stipulates that the maximum balance that can be held by an agent is N1,000,000 (*regulatory decision option* 3b).  A 2014 regulation also creates caps on transactions for Low Security Accounts, Basic Security Accounts, Moderately Secure Online Fund Transfers, and Highly Secured Online Fund Transfers, and a 2015 regulation states that outbound remittances cannot exceed US$100 per week.    More recent regulation (2017) raises the cumulative balance limits and daily cumulative transaction limits on Level 1-3 Accounts (*regulatory decision option* 3c). | Guidelines on Point-of-Sale (POS) Card Acceptance Services (2007); Circular on Introduction of Three-tiered Know Your Customer Requirements (2013); Guidelines for the Regulation of Agent Banking and Agent Banking Relationships in Nigeria (2013); Circular on Exposure Draft on the Framework for Licensing Super Agents (2014); Circular on the introduction of fees on remote-on-us ATM withdrawal transactions (2014); Circular on the Review of Operations of the NIBBSS Instant Payment (NIP) System and Other Electronic Payments Options with Similar Features (2014); Guidelines on International Mobile Money Remittance Service in Nigeria (2015); Guidelines on Mobile Money Services in Nigeria (2015); Guidelines on Operations of Electronic Payment Channels in Nigeria (2016); Review of Daily MM Wallet Transaction & BVN Requirement for Mobile Money Wallet Holders (2017) |
| Institution types that can use agents (bank/non-bank) for financial transactions | A 2007 regulation authorized CBN licensed financial and non-financial institutions to provide banking services through merchants (*regulatory decision options* 5ai and 5bi). Subsequent regulations in 2013 and 2015 reiterated that banks can engage in agent banking and mobile money initiatives. In the bank-led model, a financial institution or a consortium of financial institutions can be the lead initiator (*regulatory decision option* 5ai). Regulations in 2013 and 2015 reiterated that non-banks (but not telecommunications companies) can engage in agent banking and mobile money initiatives.  In the non-bank led model, the lead initiator must be an organization licensed by the CBN to provide mobile money services (*regulatory decision option* 5bi). A 2015 regulation states that the telecommunications company led model is not permitted in Nigeria (*regulatory decision option* 5bii). | Guidelines on Point-of-Sale (POS) Card Acceptance Services (2007); Guidelines for the Regulation of Agent Banking and Agent Banking Relationships in Nigeria (2013); Guidelines on Mobile Money Services in Nigeria (2015); Regulatory Framework for Mobile Money Services in Nigeria (2015) |
| Use of exclusive agents | Early regulation (2007) prohibited exclusivity in all areas of payment service including issuing, acquiring, processing, and sale and maintenance of hardware and software, among others. This prohibition was also reiterated in 2016 regulation.  A 2013 regulation prohibited exclusive contracts between financial institutions and agents specifically. Agents can provide services to as many financial institutions as they have capacity to accommodate (*regulatory decision option*8ci). | Guidelines on Point-of-Sale (POS) Card Acceptance Services (2007); Guidelines for the Regulation of Agent Banking and Agent Banking Relationships in Nigeria (2013); Guidelines on Operations of Electronic Payment Channels in Nigeria (2016) |
| Interoperability | Interoperability between multiple mobile money providers: Early regulation (2007) stated that all switch companies must be interoperable with the Nigeria Central Switch and Payment Terminal Service Aggregator. Regulations in 2012, 2014, 2015, and 2016 further reiterated that all mobile money operators are required to connect to the National Central Switch to ensure interoperability between platforms. One of the 2015 regulations specifically stated that mobile network operators are responsible for ensuring interconnectivity between mobile money operators. A 2016 regulation stipulates that all POS terminals must be connected to the Payments Terminal Service Aggregator and all transactions must be routed through the Payment Terminal Service Aggregator (*regulatory decision option* 4ai).  Interoperability between mobile money providers and banks: Early regulation (2007) required POS terminals to accept any card issued by any Nigerian bank and prohibited service providers from favoring any card over another. This was reiterated in a more recent (2016) regulation, which requires POS terminals to accept all cards at all merchant locations. A 2013 regulation states that there shall be an end-to-end connection from a financial institutions to its agents, in compliance with the industry standard hardware and software technology (*regulatory decision option* 4bi). | Guidelines on Point-of-Sale (POS) Card Acceptance Services (2007); Timeline for Interoperability and Interconnectivity (2012); Guidelines for the Regulation of Agent Banking and Agent Banking Relationships in Nigeria (2013); Circular on Exposure Draft on the Framework for Licensing Super Agents (2014); Regulatory Framework for Mobile Money Services in Nigeria (2015); Guidelines on Mobile Money Services in Nigeria (2015); Guidelines on International Mobile Money Remittance Service in Nigeria (2015); Guidelines on Operations of Electronic Payment Channels in Nigeria (2016) |
| Agent selection (exclusion & pre-existing requirements) | Early regulation (2007) only allowed Payments Terminal Service Providers (PTSP) licensed by the CBN to deploy, maintain and provide support for POS terminals. Criteria to become a PTSP, and performance requirements for PTSP, are to be defined by the CBN and performance is to be reviewed annually. This was reiterated in 2016. A 2013 regulation states that agents must have been in a legitimate commercial activity for a minimum of 12 months prior to applying to be an agent. It also lists the information that an entity must submit to the financial institution prior to becoming an agent. A 2014 regulation outlines the requirements that a Super-Agent must meet. Super-Agents must renew agreements with their agents every two years. A 2015 regulation requires e-payment operations to obtain CBN approval or a license from CBN. Further 2015 regulation clarifies what an agent needs to obtain a mobile money license. Additionally it states that an agent must pay a N100,000.00 non-refundable application fee to CBN and provide evidence of shareholders' fund of N2 billion. Another 2015 regulation states the requirements that institutions seeking to provide international mobile money remittance service must meet (*regulatory decision option* 7ai).    A 2013 regulation states that limited liability companies, sole proprietorships, partnerships, cooperative societies, public entities, trusts and any entity prescribed by the CBN are permitted to be agents (*regulatory decision option* 7aii). The same regulation distinguishes three agent classifications: (1) super-agents, who can oversee other agents; (2) sole-agents, who operate independently, and; (3) sub-agents, who operate under a super-agent (*regulatory decision option*7aiv) and states that faith-based or non-profit organizations, non-governmental organizations, educational institutions and bureau-de-change are not permitted to be agents.  A 2017 regulation states that card schemes and entities with management contract with a card scheme are not permitted to become PTSP, and no bank should have a controlling share in any PTSP (*regulatory decision option* 7b). | Guidelines on Point-of-Sale (POS) Card Acceptance Services (2007); Guidelines for the Regulation of Agent Banking and Agent Banking Relationships in Nigeria (2013); Circular on Exposure Draft on the Framework for Licensing Super Agents (2014); Guidelines on International Mobile Money Remittance Service in Nigeria (2015); Sanctions on Erring Banks/e-Payment Service Providers for Infractions of Payment System Rules and Regulations (2015); Guidelines on Mobile Money Services in Nigeria (2015); Guidelines on Operations of Electronic Payment Channels in Nigeria (2016) |
| Agent Services | A 2014 regulation requires all agent locations to provide CICO services for inter-scheme payments. A 2015 regulation limits transactions to the receipt of monies transmitted via mobile phones and other hand held devices to persons residing in Nigeria and foreign visitors (*regulatory decision option* 9aii).   A 2013 regulation prohibits agents from giving any guarantee, offering banking services on their own accord, providing any banking service not included in their contract with a financial institution, opening accounts, granting loans, undertaking check deposit and encashment of checks, transacting in a foreign currency, or providing cash advances (*regulatory decision option* 9b). | Guidelines for the Regulation of Agent Banking and Agent Banking Relationships in Nigeria (2013); Circular on Exposure Draft on the Framework for Licensing Super Agents (2014); Guidelines on International Mobile Money Remittance Service in Nigeria (2015) |
| Agent Reporting Requirements | Early regulation (2007) requires all industry stakeholders who process and/or store cardholder information to ensure that their terminals, applications and processing systems comply with the relevant standards. Each vendor must provide valid certificates showing compliance with these standards, and must regularly review status of all its terminals to ensure they are still compliant as standards change. These regulations were reiterated in 2016 (*regulatory decision option* 6aiii). The same 2007 regulation states that NIBSS will provide Acquirers and Payment Terminal Service Providers and their merchants the ability to view transactions and monitor performance of their devices. It states that the Payments Terminal Service Aggregator will monitor the availability and transaction traffic of all POS terminals, and will report on POS terminal performance and transaction trends to the Central Bank. A 2013 regulation stipulates that annual reports should include all agent banking operations and activities and a 2014 regulation requires Super-Agents to submit monthly information on all agent operations to the CBN, including the nature, value, and volume of transactions. Three regulations released in 2015 addressed reporting on mobile money. They require mobile money operators to include all mobile money activities in their annual reports, mobile money operators to send daily live transaction data to NIBSS in an XML format, and international mobile money remittance service providers to submit monthly to the CBN the nature, value, and volume of all transactions. The most recent regulation (2016) requires Acquirers to report on the volume and value of transactions to the CBN on a monthly basis. It also reiterates that the Payments Terminal Service Aggregator should provide reporting on POS terminal performance and transaction trends to the CBN (*regulatory decision option* 6aiii).   A 2013 regulation requires entities to submit their physical location, postal address, telephone number and working hours to the financial institution when applying to become an agent. . The financial institution is required to publish an up-to-date list of agents and their locations on its website and in its annual reports. Another regulation (2014) requires Super-Agents to provide the financial institution the names of agents and their locations (*regulatory decision option* 6ai).   A regulation from 2016 requires banks to maintain a register of all ATMS, including location, identification, and serial number of the machine. All institutions operating ATMS must file a list of all ATM locations with the CBN (*regulatory decision option* 6bi). | Guidelines on Point-of-Sale (POS) Card Acceptance Services (2007); Guidelines for the Regulation of Agent Banking and Agent Banking Relationships in Nigeria (2013); Circular on Exposure Draft on the Framework for Licensing Super Agents (2014); Guidelines on Mobile Money Services in Nigeria (2015); Guidelines on International Mobile Money Remittance Service in Nigeria (2015); Circular on the implementation of the Global Mobile Payments Monitoring and Regulation System (2015); Guidelines on Operations of Electronic Payment Channels in Nigeria (2016) |
| Agent Authentication and Due Diligence | Early regulation (2007) stated that all POS terminals must comply with standards and best practices specified by various card schemes. Vendors must provide valid certificates showing compliance with these standards. This was reiterated in more recent regulations (2016). A 2013 regulation instructs financial institutions to ensure that any institutional risk management program covers the agent-related risks. Another 2013 regulation states that the CBN should monitor the relationships between financial institutions and agents to ensure compliance with guidelines and regulations on an annual basis. 2014 regulation states that Super-Agents should submit monthly to the CBN information on the nature and number of customer complaints and remedial measures taken (*regulatory decision option* 10aii).   A 2013 regulation states that financial institutions need clear policies and due diligence processes for selecting agents, including a list of minimum standards. Financial institutions must have a signed agreement with each agent (reiterated in 2015 regulation). A 2014 regulation stipulates that applications for Super-Agent licenses should include qualifying criteria for engaging agents (*regulatory decision option* 10ai).   A 2013 regulation stipulates that agents clearly display signs which indicate that they are providing services on behalf of a financial institution, and the financial institution's contact information. Financial institutions should provide a communication channel for customers and agents to lodge complaints. This channel can also be used to verify the authenticity and identity of an agent, its physical location and the validity of its agent banking business. A subsequent 2014 regulation also states that customers should be provided a channel, such as web, SMS, USSD short-code, etc., through which they can verify the authenticity of an agent (*regulatory decision option* 10b). | Guidelines on Point-of-Sale (POS) Card Acceptance Services (2007); Guidelines for the Regulation of Agent Banking and Agent Banking Relationships in Nigeria (2013); Circular on Introduction of Three-tiered Know Your Customer Requirements (2013); Circular on Exposure Draft on the Framework for Licensing Super Agents (2014); Circular on the implementation of the Global Mobile Payments Monitoring and Regulation System (2015); Guidelines on Operations of Electronic Payment Channels in Nigeria (2016) |
| Different Agent Classes | Early regulations (2007) state rules for the following classes: merchant acquirers; payment terminal service providers; PoS terminal owners; payments terminal service aggregators; card issuers; merchants; card associations and card schemes; and switching companies (*regulatory decision option* 11). These classes are affirmed in 2016 regulations.  2013 regulations describe three agent classifications: super-agents (they can oversee other agents) (i); sole-agent (independent agent); and (iii) sub-agents (operate under a super-agent) (*regulatory decision option* 11). 2014 further stipulate super-agents, which oversee agents, to have a minimum of 50 agents to be classified as a super-agent. | Guidelines on Point-of-Sale (POS) Card Acceptance Service (2007); Guidelines for the Regulation of Agent Banking and Agent Banking Relationships in Nigeria (2013); Circular on Exposure Draft on the Framework for Licensing Super Agents (2014); Guidelines on Operations of Electronic Payment Channels in Nigeria (2016) |
| Agent e-float | No relevant regulations were found on this topic. |  |
| Agent Compensation | No relevant regulations were found on this topic. |  |

## Table S46. Summary of cash-in, cash-out (CICO) regulation in Pakistan.

| **Type of regulations** | **Description** | **Regulation** |
| --- | --- | --- |
| Know Your Customer Requirements | Regulation issued in 2016 states that for level 2 accounts, the highest class of accounts, institutions must fulfill all KYC requirements specified under AML/CFT Regulations and Guidelines issued by SBP (*regulatory decision option* 1ai). However, regulation issued in this same year outlines less stringent KYC procedures for certain accounts (*regulatory decision option* 1bi). For level 0 accounts, customers must provide a verified SIM card, their CNIC, the institution must confirm customer information with NADRA, and the institution must capture and retain a photo of the CNIC and of the customer. For level 1/biometric accounts, in addition to level 0 requirements the institution must also confirm the customer's cell phone number. When opening an account, all customers must provide at a minimum: a) full name; b) CNIC/passport/NICOP/POC/ARC number; c) existing residential address, telephone numbers, and email; d) date of birth; e) nationality or place of birth; and f) source of earnings (*regulatory decision option* 1bi). | Branchless Banking Regulations (2016); Framework for Branchless Banking Agent Acquisition and Management (2016); Anti-Money Laundering and Combating the Financing of Terrorism (AML/CFT) Regulations for Banks & DFIs (2016) |
| Interoperability | A 2016 regulation states that that third party service providers (TPSPs) should have arrangements with peer institutions to allow for switching and routing of inter-bank mobile banking transactions (*regulatory decision options* 4ai & 4bi). The most recent regulation (2017) reaffirm this, stating that the licensee should be capable of switching and routing all interbank Wallet-to-Wallet and Wallet-to-bank account fund transfers from the branchless bank issuer to the acquirer through an Authorized Payment Service Operator (*regulatory decision options* 4ai & 4bi). Regulation aims to provide a level playing field for financial institutions and non-banks by introducing a neutral, third party model in which financial institutions and their partners can join together to create a sustainable mobile banking ecosystem. A third party service provider (TPSP) shall have arrangements with peer TPSPs for clearing, processing, routing and switching electronic transactions and inter-bank mobile banking transactions. TPSPs shall make available its network to all parties in the arrangement, based on criteria that is transparent and generally applicable to all institutions without discriminatory practices. | Branchless Banking Regulations (2016); Framework for Branchless Banking Agent Acquisition and Management (2016); Regulations for Technical Implementation of Mobile Banking (2016); Third Party Service Provider (TPSP) Licensing (2017) |
| Institution types that can use agents (bank/non-bank) for financial transactions | A 2016 regulation states that only bank-led models of branchless banking will be permitted in Pakistan. However, banks are able to implement branchless banking through either agency arrangements or through creating a joint-venture between a financial institution and a telecom operator/non-bank (*regulatory decision option* 5ai).  All telecom operators having a valid license shall be eligible/entitled to offer technical services to authorized financial institution for provision of mobile banking services without obtaining a separate license for this purpose from PTA under one-to-one model. Mobile phone banking can be implemented using one-to-one, one-to-many, and many-to-many models. | Branchless Banking Regulations (2016); Regulations for Mobile Banking Interoperability (2016); Regulations for Technical Implementation of Mobile Banking (2016) |
| Use of exclusive agents | Two separate 2016 regulations indicate that one agent may provide services for and have arrangements with multiple banks as long as the agents have separate agreements with each (*regulatory decision option* 8bi). The one-to-one model (where a bank partners with a telecom operator or non-bank) does not necessarily require exclusivity. Therefore, one bank can have several one-to-one arrangements with many telecom operators or non-banks, or alternatively one telecom operator or non-bank can have several one-to-one arrangements with many banks, provided that such arrangements are under proper agency/service level agreements. One agent can provide services to multiple financial institutions (FI) provided the agent has a separate Service Level Agreement with each FI. Alternately, FIs may organize their agent network using open architecture so that the agents may serve other FIs’ customers using infrastructure provided by one FI. | Branchless Banking Regulations (2016); Framework for Branchless Banking Agent Acquisition and Management (2016) |
| Agent selection (exclusion & pre-existing requirements) | Technology service providers do not face specific restrictions to become branchless banking agents, provided they meet the general criteria for becoming an agent. Regulation in 2016 stipulates that when establishing a new agent, a financial institution (FI) must establish procedures that define: various agent categories or the agent structure based on the types of branchless banking (BB) services they are allowed to offer; minimum selection criteria for each category of agent including business experience and financial position; and individuals/businesses eligible to provide BB services per the FI's policy (*regulatory decision option* 7ai). | Framework for Branchless Banking Agent Acquisition and Management (2016) |
| Agent Compensation | Regulations in 2016 stipulate the fee and revenue sharing structure shall be included in the Service Level Agreement contract (regulatory option 2d). The responsibility for supplying up-front and operational costs of branchless banking operations needs to be defined in the SLA as well. | Framework for Branchless Banking Agent Acquisition and Management (2016) |
| Agent Services | Agents should not offer any branchless banking services other than those specified in the agency agreement. The agent will not perform management functions, make management decisions, open level 2 accounts, or act or appear to act in the capacity equivalent to that of a member of management or an employee of the financial institution (*regulatory decision option* 9b). | Branchless Banking Regulations (2016); Framework for Branchless Banking Agent Acquisition and Management (2016) |
| Agent e-float | Regulations in 2016 state that contracts between a financial institutions and agents must specify suitable limits for cash holdings (*regulatory decision option* 9ai). The financial institution may devise a policy whereby agents shall be required to maintain sufficient cash on hand and money in their branchless banking accounts to provide uninterrupted services to customers. In all branchless banking models, the customer account must reside with some financial institution, each transaction must hit the actual customer account, and no actual monetary value may be stored on the mobile-phone or technology service provider's service. | Branchless Banking Regulations (2016); Framework for Branchless Banking Agent Acquisition and Management (2016) |
| Different Agent Classes | A 2016 regulation outlines permissible agent models, including: Super Agents (well-established owned or franchised retail outlets, or a distribution setup; responsible for managing and controlling subagents; may include fuel distribution companies, Pakistan Post, courier companies, chain stores etc.); Direct Agents (may include large- to medium-sized stores etc., which have a separate agency/Service Level Agreement with the financial institutions); Sub Agents (branches/outlets or franchised locations managed by a super-agent and not directly controlled by the financial institutions on a day-to-day basis)(*regulatory decision option* 11). Additionally agents may operate one of the following models: One-to-one (where a bank partners with a telecom operator or non-bank); one-to-many (where a bank offers branchless banking through the mobile connection of any telecom operator); and many-to-many (where many banks and many telecom operators or non-banks jointly offer services to all bankable customers). | Branchless Banking Regulations (2016); Framework for Branchless Banking Agent Acquisition and Management (2016) |
| Caps & Fees | Regulation from 2016 stipulates that charge, fee, and income sharing structures must be agreed upon among the among the authorized financial institution, third party service providers, and telecom operator in a transparent manner prior to operation (*regulatory decision option* 2ai). This structure should be commensurate with the mobile banking services standards and disclosed to the consumers.  A 2016 regulation stipulates that the maximum balance permitted on a level 0 account is Rs. 200,000; and for a level 1/biometric account it is Rs. 400,000 (*regulatory decision option* 3a). Regulation from this year also outlines maximum daily, monthly, and yearly transaction amounts for level 0 and level 1 accounts (*regulatory decision option* 3c). limits for level 0 accounts are lower than those for level 1. Maximum balance: Rs. 200,000 for level 0; 400,000 for level 1/biometric. Maximum transactions: Rs. 25,000 per day, 40,000 per month, and 200,000 per year for level 0; 50,000 per day, 80,000 per month, and 800,000 per year for level 1/biometric; the financial institution can determine limits for level 2 account; 50,000 per month for account-to-person (non-accountholders) or person (non-accountholders)-to-IBFT transfers with bio-verification and 25,000 without; and 50,000 per month for person-to-person (non-accountholders) with bio-verification and 15,000 without.   Financial institutions are able to determine their own limits for level 2 accounts. Contracts between a financial institution and their agents must specify all limits. | Branchless Banking Regulations (2016); Framework for Branchless Banking Agent Acquisition and Management (2016); Regulations for Mobile Banking Interoperability (2016) |
| Agent Reporting Requirements | A 2016 regulation stipulates that financial institutions must provide information on its branchless banking agent network to SBP, including the full name of the business owner and their contact details, including their business address (*regulatory decision option* 6ai). The following minimum information may be obtained and verified by the financial institution (FI) for each agent: full name of business owner and CNIC and contact details; integrity, personal qualities, and reputation of the business owner; financial position/net worth and credit profile of the owner; knowledge, experience, capability, and competency of the potential agent to conduct agent banking services as an acceptable quality level; ability of the agent to control operational risks related to agent banking; information of manager of the shop/outlet and level of education of the owner;  details of persons at agents shop, who shall provide branchless banking (BB) services to the customers; business address; and nature of commercial activity of the business and ongoing status. To ensure their focus on expanding and creating new retail footprint, geographical spread and overall BB outlet density, FIs shall formulate policy on the sharing of BB agents with other FIs. However, FIs shall follow the limits/percentage on BB agent sharing as defined by State Bank of Pakistan (SBP) from time to time. | Framework for Branchless Banking Agent Acquisition and Management (2016) |
| Agent Authentication and Due Diligence | Regulation from 2016 stipulates that financial institutions are responsible for having clear, well documented Authentication and Due Diligence policies, including initial due diligence (*regulatory decision option* 10ai). The following minimum information should be obtained for all agents: full name of business owner and CNIC and contact details; integrity, personal qualities, and reputation of business owner; financial position/net worth of owner; knowledge, experience, capability, and competency of the potential agent; and ability of the agent to control operational risks. 2016 regulations stipulate that as part of the application process for branchless banking services, financial institutions must include information on agent liquidity management procedures. Additionally, financial institutions must ensure that the scope and coverage of their internal audit function has been expanded to commensurate with complexity and risks inherent in agent activities. Financial institutions are responsible for having clear, well documented Authentication and Due Diligence policies, including regular due diligence checks to be performed at specific intervals (*regulatory decision option* 10aii). The State Bank of Pakistan (SBP) shall carry out inspection and/or diagnostic studies of branchless banking service providers and their agents (*regulatory decision option* 10aii). | Branchless Banking Regulations (2016); Framework for Branchless Banking Agent Acquisition and Management (2016); Regulations for Mobile Banking Interoperability (2016) |
| Other: Alternative Services of Branchless Banking | Permissible models include: 1. Using technologies not limited to mobile phone, 3G/4G spectrum, GPRS, POS terminals and internet banking etc.; 2. Issue ATM/debit card for branchless banking customers (domestic transactions only); and 3. Offer international transaction facility on ATM/debit cards to Level 2 account holders. | Branchless Banking Regulations (2016) |
| Other: Location of cash-in and cash-out | Customers can use a variety of options including bank-branch counters, ATM machines, and authorized agent locations. | Branchless Banking Regulations (2016) |
| Other: Opening account remotely | Account shall be opened against verified SIM Card. Customer shall visit bank branch / agent for initial cash deposit. Mobile number shall remain in the name of same person, who is requesting to open the account. In line with National Financial Inclusion Strategy to promote financial inclusion in the country, it has been decided to allow opening of remote accounts for Level 0 customers. | Branchless Banking Regulations (2016) |
| Other: Security | Financial institutions shall design the system to automatically stop the transaction if tried beyond the assigned limit. | Framework for Branchless Banking Agent Acquisition and Management (2016) |

## Table S47. Summary of cash-in, cash-out (CICO) regulation in Tanzania.

| **Type of regulations** | **Description** | **Regulation** |
| --- | --- | --- |
| Know Your Customer Requirements | A 2007 regulation states that the bank or financial institution shall at minimum ensure effective Know Your Customer principles be applied using reliable methods for verifying the identity and authorization of new customers, as well as authenticating the identity and authorization of established customers seeking to initiate electronic transactions and conduct business over the internet (*regulatory decision options* 1ai and 1aii). In 2010, a regulation stated that an approved banking institution should ensure that agents identify customers with at least two factor authentications like IDs, PINs, passwords, ATM card, secret code or secret message while performing any transaction requiring a transaction (*regulatory decision option* 1aii). In 2015, two regulations stated more specifically that electronic money issuers shall comply with all Anti-Money Laundering and Combating the Financing of Terrorism identification requirements for all mobile money and cash-in transactions (*regulatory decision option* 1aii), and one of the 2015 regulations also stated that all mobile money and cash-in transfers require a registered phone number; and all cash-out transfers require a verified ID (*regulatory decision option*1aii). The requirement to comply with two-factor authentication for transactions was reinforced in a 2017 regulation (*regulatory decision option* 1aii). | Electronic Payment Schemes Guidelines (2007); Guidelines on Agent Banking for Banking Institutions (2013); Government Notice No. 245, The Foreign Exchange Act, Cap. 271 (2015); The Payment Systems (Electronic Money) Regulations (2015); Guidelines on Agent Banking for Banks and Financial Institutions (2017) |
| Interoperability | A 2007 regulation states that electronic payment schemes shall be open systems capable of becoming interoperable with other payment systems in the country and shall comply with minimum international acceptable standards (*regulatory decision options* 4ai & 4bi). | Electronic Payment Schemes Guidelines (2007) |
| Institution types that can use agents (bank/non-bank) for financial transactions | A 2013 regulation allows banks to conduct business through agents (*regulatory decision option* 5ai). One 2015 regulation allows electronic money issuers to conduct business through agents (*regulatory decision option* 5bi). A different 2015 regulation states that Class B bureau de change entities may conduct money transfer only through banks, financial institutions, mobile network operators, or international money transfer agents (*regulatory decision options* 5ai & 5bi). A 2017 regulation reinforced the 2013 regulation, allowing banks to conduct business through agents (*regulatory decision option* 5ai). | Guidelines on Agent Banking for Banking Institutions (2013); The Payment Systems (Electronic Money) Regulations (2015); Government Notice No. 245, The Foreign Exchange Act, Cap. 271 (2015); Guidelines on Agent Banking for Banks and Financial Institutions (2017) |
| Use of exclusive agents | A 2013 regulation states the contracts between agents and financial institutions may not be exclusive. This was reinforced in both 2015 and 2017 regulations (*regulatory decision option* 8ci) | Guidelines on Agent Banking for Banking Institutions (2013); The Payment Systems (Electronic Money) Regulations (2015); Guidelines on Agent Banking for Banks and Financial Institutions (2017) |
| Agent selection (exclusion & pre-existing requirements) | A 2013 regulation states that a person intending to be appointed as an agent is required to have operated a lawful commercial activity for at least two years preceding the date of the application to become an agent and such commercial activity must be ongoing; this regulation also lists the types of entities that may serve as agents (*regulatory decision option* 7ai). This regulation also states that entities cannot become agents if their sole activity is agent banking (*regulatory decision option* 7b). A 2017 reinforces the 2013 regulation, but shortens the amount of time operating a commercial activity from two years to 18 months (*regulatory decision option* 7ai). | Guidelines on Agent Banking for Banking Institutions (2013); Guidelines on Agent Banking for Banks and Financial Institutions (2017) |
| Agent Compensation | A 2017 regulation states that written agreement between agent and the financial institution/bank must mention the set fees or revenue sharing structure (*regulatory decision option*2d). | Guidelines on Agent Banking for Banks and Financial Institutions (2017) |
| Agent Services | A 2013 regulations stipulates that an agent shall not: 1) carry out transactions in currencies other than Tanzanian shillings; 2) offer guarantees to bank clients; or 3) open accounts, grant loans or advances, or carry out any appraisal functions. A 2015 regulation adds that agents are not allowed to send person-to-person payment transfers. A 2017 regulation adds 13 additional restricted activities (*regulatory decision option*9b). | Guidelines on Agent Banking for Banking Institutions (2013); The Payment Systems (Electronic Money) Regulations (2015); Guidelines on Agent Banking for Banks and Financial Institutions (2017) |
| Agent e-float | A 2015 regulation established maximum daily balance (float) of 100,000,000 Tanzania Shillings (*regulatory decision option*9ai). | The Payment Systems (Electronic Money) Regulations (2015) |
| Different Agent Classes | Agent classes include: Tier I; Tier II; Small and Medium Enterprises (Tier III); Retail Agents (Tier IV); Super Agents; and Large Businesses (*regulatory decision option* 11). Additionally there are two classes of bureaus de change: class A and class B. | The Payment Systems (Electronic Money) Regulations (2014); Excise Duty on Charges and Fees (2015) |
| Caps & Fees | A 2013 regulation stipulates that agents may not charge fees to customers (*regulatory decision option* 2bii). A 2015 regulation provides maximum caps on both balances and transactions, and differentiates these caps based on tier 1, tier 2, and small business/enterprise (*regulatory decision option* 3a). A 2017 regulation requires that agents and the financial institution/bank must mention the set fees or revenue sharing structure within the agreement (*regulatory decision option* 2ai).   A 2014 requires that banks, financial institutions, and telecommunications service providers pay the Tanzania Revenue Authority 10% of the total amount of money collected from customers as charges or fees. | Guidelines on Agent Banking for Banking Institutions (2013); Excise Duty on Charges and Fees (2014); The Payment Systems (Electronic Money) Regulations (2015); Guidelines on Agent Banking for Banks and Financial Institutions (2017) |
| Agent Reporting Requirements | A 2013 regulation states that banks and financial institutions must publicize a list of all their agents and locations (*regulatory decision option* 6ai). A 2015 regulation states that electronic money issuers shall maintain and submit to the Bank of Tanzania records of appointed agents that shall include agents and addresses and GPS coordinates; the Bank of Tanzania may then develop a public registry of agents to share online (*regulatory decision option* 6ai). A separate 2015 regulation states that Class A bureau de change entities shall submit to the Bank of Tanzania a certified copy of their lease agreement or title deed of the premises on which the business will be conducted (*regulatory decision option* 6ai). Finally, a 2017 regulation permits an approved bank or financial institution to carry out periodic physical visits to ensure that agents operate strictly within the requirements of the law, guidelines, and the agent agreement; an approved bank or financial institution shall report its agent banking activities to the Bank in the form and frequency prescribed by the Bank (*regulatory decision options* 6ai and 6aiii). | Guidelines on Agent Banking for Banking Institutions (2013); The Payment Systems (Electronic Money) Regulations (2015); Government Notice No. 245, The Foreign Exchange Act, Cap. 271 (2015); Guidelines on Agent Banking for Banks and Financial Institutions (2017) |
| Agent Authentication and Due Diligence | A 2007 regulation states that banks and financial institutions shall at minimum establish formal policies and procedures for identifying appropriate methodologies to ensure agents are properly authenticated (*regulatory decision option* 10aii). A 2013 regulation states that an approved banking institution shall conduct assessment and due diligence (Know-Your-Agent) on the business owner and business operations to ensure that an agent is well established, having good reputation and enjoying the confidence of the community (*regulatory decision option* 10ai). In 2014 regulation states that a bureau de change entity shall not commence business until the business premises, security facilities, communication facilities, processing equipment and accounting systems are in place and have been inspected, reviewed, and approved by the Bank of Tanzania (*regulatory decision option* 10ai); this regulation also requires that bureau de change entities display a copy of the valid license in a conspicuous place at each of its premises (*regulatory decision option* 10b). In 2015, a regulation states that electronic money issuers shall, prior to appointing agents, submit to the Bank of Tanzania documentation of the proposed agents, which includes procedures for appointing agents as well as an ongoing a due diligence plan (*regulatory decision options* 10ai and 10aii). A 2017 regulation states that an approved bank or financial institution shall put in place clear, well documented agent Due Diligence policies and procedures. The procedures shall contain, at a minimum new agent take-on procedures and initial due diligence (*regulatory decision option* 10ai) as well as regular due diligence checks to be performed in intervals (*regulatory decision option* 10aii). | Electronic Payment Schemes Guidelines (2007); Guidelines on Agent Banking for Banking Institutions (2013); Excise Duty on Charges and Fees (2014); The Payment Systems (Electronic Money) Regulations (2015); Guidelines on Agent Banking for Banks and Financial Institutions (2017) |

## Table S48. Summary of cash-in, cash-out (CICO) regulation in Uganda.

| **Type of regulations** | **Description** | **Regulation** |
| --- | --- | --- |
| Know Your Customer Requirements | In 2013, Uganda required the entity conducting customer verification should require at least one of the following documents to verify the identity of the customer: a valid passport, driving permit, identity card, voter’s card, financial card, local administration letter or business registration certificates (*regulatory decision option* 1a). Additionally, every transaction shall require authentication by a customer’s PIN. Recent regulations (2017) add that financial institutions shall ensure that every transaction requires at least two-factor authentication (*regulatory decision option*1a). | Mobile Money Guidelines, 2013 (2013); The Financial Institutions (Agent Banking) Regulations (2017) |
| Interoperability | Early regulations (2013) stated that mobile money service providers shall utilize systems capable of becoming interoperable with other payment systems in the country and internationally, in order to facilitate full interoperability (*regulatory decision options* 4ai & 4bi). This agreement must not contain exclusivity clauses. Mobile money service providers may establish partnerships with multiple licensed institutions and vice versa. | Mobile Money Guidelines, 2013 (2013) |
| Institution types that can use agents (bank/non-bank) for financial transactions | 2016 regulation stated that a person licensed to carry out financial institution business may carry out the licensed business through an agent (*regulatory decision option* 5bi). Recent regulations (2017) state that with written approval by the Central Bank, a financial institution (FI) can conduct agent banking. An FI includes: a commercial bank, merchant bank, mortgage bank, post office savings bank, credit institution, a building society, an acceptance house, a discount house, a finance house, an Islamic financial institution or any institution which by regulations issued under the Act is classified as a financial institution by the Central Bank (*regulatory decision options* 5ai & 5bi). | The Financial Institutions (Amendment) Act (2016); The Financial Institutions (Agent Banking) Regulations (2017) |
| Use of exclusive agents | 2013 regulation stated that the mobile money service provider selects and manages mobile money agents, and the agent agreement should not provide for exclusivity (*regulatory decision option* 8ci). 2017 regulation specifies that the agreement between a financial institution and an agent shall not include a provision prohibiting the agent from conducting agent banking with other financial institutions (*regulatory decision option* 8ci). An agent may provide agent banking for other approved financial institutions provided that a) the agent has entered into an agency agreement with each financial institution and b) the agent has the capacity to manage the transactions for the different financial institutions (*regulatory decision option* 8ci). | Mobile Money Guidelines, 2013 (2013); The Financial Institutions (Agent Banking) Regulations (2017) |
| Agent selection (exclusion & pre-existing requirements) | Recent regulation (2017) stipulates that a person appointed as an agent must: 1) operate an account at a financial institution licensed by the Central Bank for consecutive six months prior to the application; 2) have a licensed business; 3) have a physical address; 4) have adequate and secure premises; and 5) been engaged in the licensed business for at least 12 months (*regulatory decision option* 7ai). 2017 regulations also state that a person shall not be appointed as an agent unless that person is: 1) a sole proprietorship; 2) a partnership; 3) a company; 4) a cooperative society; 5) a microfinance institution; or 6) an entity approved by the Central Bank (*regulatory decision option* 7aii). Recent regulations (2017) further state that a financial institution shall not conduct agent banking with its employees, affiliates, or associates (*regulatory decision option* 7b). | The Financial Institutions (Agent Banking) Regulations (2017) |
| Agent Compensation | 2013 regulations stated that agency commission should be paid to the agents by the mobile money service provider (*regulatory decision option* 2d). Recent regulations (2017) state that a financial institution shall compensate agents for the services rendered, as per the contract (*regulatory decision option* 2d). If the financial institution is a mobile money service provider, this provider will be responsible for paying commission to the agent. | Mobile Money Guidelines, 2013 (2013); The Financial Institutions (Agent Banking) Regulations (2017) |
| Agent Services | Recent regulations (2017) state that an agent shall not use as part of its name, words like "bank," "financial institution," "financial intermediary," or their derivatives or any word suggesting that the agent is itself a financial institution (*regulatory decision option* 9b). Furthermore, an agent shall not: a) offer financial institution businesses on its own accord except where it is the agent's principal business at the time of engagement; b) continue with the agency banking with criminal record; c) provide or hold out to be providing any banking service that is not in the agreement; d) operate a transaction when the system is down, there is a communication failure, or the customer is absent; e) carry out a transaction when a receipt indicates the transaction cannot be generated; f) charge fees directly to customers; g) undertake check deposits or encashment; h) distribute checkbooks; i) distribute debit cards, credit cards, or PIN mailers; j) conduct foreign exchange transactions; k) subcontract other person to provide agency banking services; l) provide agency banking at a location other than the physical agent address; m) open accounts, grant loans or advances, or carry out appraisal functions except as may be permitted by law; or n) be a guarantor to the financial institution's clients (*regulatory decision option* 9b). | The Financial Institutions (Agent Banking) Regulations (2017) |
| Agent e-float | No relevant regulations were found on this topic. |  |
| Different Agent Classes | No relevant regulations were found on this topic. |  |
| Caps & Fees | Early regulations (2013) stated that the agent shall clearly display in a conspicuous place: 1) All applicable charges and fees for the mobile money service and 2) a written notice that no charges or fees are levied at the agent location (*regulatory decision option* 2ai). 2013 regulations also stated mobile money agent shall not charge any fees directly to the customers and limits should be set for frequency, volume, and value of transactions; and these limits, as well as any revisions thereof, shall be sent to Bank of Uganda for approval (*regulatory decision option* 2bii & 3c). Recent regulations (2017) state that an agency agreement shall set out transaction limits of the agent (*regulatory decision option* 3d). | Mobile Money Guidelines, 2013 (2013); The Financial Institutions (Agent Banking) Regulations (2017) |
| Agent Reporting Requirements | Early regulations (2013) stated that mobile money services must report the number of agents and the agents' balances each month (*regulatory decision option* 6aiii). Recent regulation (2017) states that a financial institution shall provide monthly reports to the Central Bank on new and exited agents (*regulatory decision options* 6ai & 6aiii). | Mobile Money Guidelines, 2013 (2013); The Financial Institutions (Agent Banking) Regulations (2017) |
| Agent Authentication and Due Diligence | Early regulations (2013) stated that a mobile money service provider in its dealings with mobile money agents, must put in place an effective agent selection process and carry out due diligence on its agents (*regulatory decision option* 10ai & 10b). The agent shall clearly display in a conspicuous place the agent’s unique identification number provided by the mobile money service provider. Recent regulation (2017) adds on that a financial institution shall ensure that the agent clearly displays in a conspicuous place at its premises of operation the signage of the financial institution responsible for the agent and the agent's unique ID number and a telephone line that the customers can access (*regulatory decision option* 10b). Additionally, 2017 regulations state that The Central Bank has power to: a) request for any information from an agent at any time where it deems it necessary; b) carry out an examination of an agent; c) appoint an appropriate professional at the cost of the financial institution, to conduct a special audit on an agent banking service; d) direct an agent to take any action or cease from any conduct as it may be deemed necessary; e) direct the termination of an agency agreement or closure of an agency business as it deems it fit; or f) direct a financial institution to take measures against an agent (*regulatory decision option* 10aii). | Mobile Money Guidelines, 2013 (2013); The Financial Institutions (Agent Banking) Regulations (2017) |
| Other: Cash out by unregistered mobile banking users | All funds sent to unregistered users and are not cashed out within two weeks must be returned to the sender | Mobile Money Guidelines, 2013 (2013) |
