# Supplementary material for: Mobile money and branchless banking regulations affecting cash-in, cash-out networks in low- and middle-income countries
Source: Gates Open Res. 2018 Nov 28;2:64. [Version 1] doi: 10.12688/gatesopenres.12876.1 (PMC6610045; doi:10.12688/gatesopenres.12876.1)
Supplement: Supplementary file 5 [file gatesopenres-2-13965-s0004.tgz › c81843c3-9735-4fc0-b220-1bb80f79d004_Supplementary_File_5_Agent_Exclusivity_and_Market_Growth.docx]

**Supplementary File 5: Agent Exclusivity Regulations and Cash-in Cash-out (CICO) Market Growth**

This addendum further explores the implications of agent exclusivity regulations – either requiring or prohibiting agents to work with a single provider – on cash-in, cash-out (CICO) market growth. Tarazi & Breloff (2011) argue that regulations allowing or mandating agent exclusivity may encourage early market growth, but later limit competition in the market for CICO services. In a similar vein, Muthiora (2015) contends that finding, training, and supervising a network of agents is a heavy investment for the first CICO entrant, which exclusivity regulations protect. A systematic search, however, revealed no published evidence of the impacts of agent exclusivity regulations, though we find several other authors who speculate similar effects (Bourreau & Valetti, 2016; Houpis & Bellis, 2007; Castri, 2013).

In the absence of a counterfactual (i.e., we do not have data on CICO activity under a different set of regulations) hypotheses around national regulatory effects are difficult to test. Nonetheless, we explore the timeline of exclusivity regulations alongside DFS adoption rates and mobile money deployment data as a proxy for market growth and competition (Table S5A). We compare Bangladesh, Indonesia, India, and Pakistan, where agent exclusivity is mandated or allowed, to Kenya, Nigeria, Tanzania, and Uganda, where agent exclusivity has been prohibited since 2013. We consider trends in market growth and competition in each country before and after relevant regulations were introduced and ultimately find no simple patterns associating agent exclusivity with market growth.

Countries where agent exclusivity is allowed or mandated

*Bangladesh*

Sub-agents were required to be exclusive in 2013 and all agents were required to be exclusive in 2017. Using the Financial Inclusion Index (FII) data from 2013 to 2016, we see that the percentage of mobile money users among Bangladeshis increased from 22% in 2013 to 23% in 2014, 33% in 2015, and 40% in 2016 (see Table S5a). Using the same FII data, we find that the percentage of “digitally included” Bangladeshis decreased from 20% in 2013 to 15% in 2014, increased to 18% in 2015, and remained at 18% in 2016. Using GSMA’s mobile money deployment tracker, we observe 1 deployment in 2006, 2009, and 2010; 3 in 2011 and 2012; 2 in 2013 and 2014; and 0 each year thereafter. Most of the market growth occurred prior to the 2013 regulation and no new companies have deployed since the 2017 regulation.

*India*

Sub-agents were required to be exclusive in 2013. Using the FII data from 2013 to 2016, we see the percentage of Indonesians who were “digitally included” decrease from 47% in 2013 to 37% in 2014, increase to 49% in 2015, and decrease to 30% in 2016 (see Table S5a). Using the same FII data, we find that the percentage of mobile money users among Indians was at 0% in 2013 and 2014 and 1% in 2015 and 2016. Using GSMA data, we observe 1 deployment in 2007, 1 in 2010, 4 in 2012, 4 in 2013, 1 in 2014, 1 in 2015, and 0 for each year thereafter. We do observe a significant increase in market growth and competition the year prior and the year of the exclusivity regulation.

*Indonesia*

Agents were required to be exclusive in 2014. Using the FII data from 2013 to 2016, we see an increase in the percentage of Indonesians who were “digitally included” from 0% in 2013 to 20% in 2014, and another increase to 23% in 2015. There was a decrease from 2015 to 2016 of 1% (23% in 2015 down to 22% in 2016), see Table S5a for detail. Using GSMA data, we observe 1 mobile money deployment in 2007, 0 from 2008 to 2011, 2 in 2012, 4 in 2013, and none thereafter. The FII data from Indonesia are consistent with a prediction that agent exclusivity encourages early market growth but limits future competition, but this does not constitute evidence. There are only four years of data available and the FII’s “digital inclusion” measure is very broad (compared to a measure of digital financial services adoption) and may be capturing confounding variables. The GSMA data run counter to a hypothesis that mandating agent exclusivity encourages early market growth (although competition is observed to be limited in the long-run). Similar to India, we observe a significant increase in market growth the year prior to the exclusivity regulation.

*Pakistan*

Agents continue to be allowed to practice exclusivity in Pakistan. The only regulation related to agent exclusivity was created in 2016 and states that agents can work for multiple financial institutions as long as they have separate contracts with each. Using the FII data from 2013 to 2016, we see a relatively stable percentage of Pakistanis who were “digitally included” (ranges between 7% and 9%) and “mobile money users” (ranges between 7% and 9%). The GSMA data show an average of 1 mobile money deployment per year from 2009 to 2013 and no deployments thereafter. While the FII data do not show any trends, the GSMA data are consistent with the idea that allowing agent exclusivity may encourage early market growth, but later limit competition in the market for CICO services.

Countries where agent exclusivity became prohibited

*Kenya*

Safaricom often used exclusivity clauses in contracts with their M-Pesa agents until it was banned *de jure*for banks in 2010 by the Central Bank of Kenya and for all financial institutions in 2014 by the Competition Authority of Kenya (Mazar, Pillai, & Staschen, 2016). The data on “mobile money transfer subscriptions” collected by the Communications Authority of Kenya (CAK) marginally supports the hypothesis that mandating agent interoperability limits early market growth but later promotes competition. Subscriptions increased by 0.5 million from 2014 to 2015, decreased by almost 2 million from 2015 to 2016, and then increased by 15 million from 2016 to 2017 (see Table S5a). On the other hand, GSMA data of mobile money deployments show no obvious trends in competition before and after the 2011 and 2014 regulations.

*Nigeria*

Exclusivity between financial institutions and agents was banned in 2013. The FII data show that the percentage of Nigerians who were “digitally included” fluctuated within the range of 32% to 40% from 2013 to 2016. The FII Data also show that the percentage of Nigerians who were mobile money users stayed under 2% for the same time range. The GSMA data show 9 mobile money deployments in 2011, 7 in 2012, none in 2013 (the year of the regulation), 2 in 2014, and none thereafter. In India and Indonesia, we observe a significant increase in market growth the year prior to allowing agent exclusivity regulation, and in this case, an increase in growth prior to banning it.

*Tanzania*

Regulations banning exclusivity among agents of non-banking institutions were passed in Tanzania in 2015. Prior to that, in 2014, three leading operators (Tigo, Airtel, and Zantel) announced an agreement to send and receive mobile money with users of rival services (Bourreau & Valetti, 2016). In Tanzania, smaller players organized to become agent interoperable to compete with a larger market player, despite the allowance of agent exclusivity at the time.

There is only one year of FII data since the 2014 regulation (see Table S5a) and the GSMA data show an average of 1 deployment per year from 2006 to 2013, 1 deployment in 2014, 0 in 2015, 1 in 2016, and 0 in 2017.

*Uganda*

Agent exclusivity was prohibited between mobile service providers and agents in 2013. FII data show that the percentage of Ugandans who were “digitally included” increased from by 4% from 2013 to 2014, by 1% from 2014 to 2015, and by 1% in 2016. FII data also show that the percentage of Ugandans who were mobile money users stayed the same from 2013 to 2014, increased by 4% from 2014 to 2015, and increased by 6% from 2015 to 2016. GSMA data show an average of 1 deployment per year from 2009 to 2017 and no obvious trends in competition.

**Table S5a. Agent exclusivity regulations timeline & digital financial service data**

| **Country** | **Year  & any relevant exclusivity regulations** | **Digitally Included, FII Index (% of people)** | **Not digitally included, FII Index (% of people)** | **Mobile Money User, FII Index (% of people)** | **Mobile Money Non-User, FII Index (% of people)** | **Number of mobile money transfer subscriptions (in millions)** | **Number of mobile money deployments** |
| --- | --- | --- | --- | --- | --- | --- | --- |
| **Bangladesh** | 2010 | NA | NA | NA | NA | NA | 1 |
|  | 2011 | NA | NA | NA | NA | NA | 3 |
|  | 2012 | NA | NA | NA | NA | NA | 3 |
|  | 2013  Agents may work for more than one bank, but sub-agents can only work for one | 20 | 80 | 22 | 78 | NA | 2 |
|  | 2014 | 15 | 85 | 23 | 77 | NA | 2 |
|  | 2015 | 18 | 82 | 33 | 67 | NA | 0 |
|  | 2016 | 18 | 82 | 40 | 60 | NA | 0 |
|  | 2017  All agents cannot enter contracts with more than one bank. | NA | NA | NA | NA | NA | 0 |
| **Indonesia** | 2010 | NA | NA | NA | NA | NA | 0 |
|  | 2011 | NA | NA | NA | NA | NA | 0 |
|  | 2012 | NA | NA | NA | NA | NA | 2 |
|  | 2013 | 0 | 0 | NA | NA | NA | 4 |
|  | 2014  Agents may only partner with one bank or service provider. | 20 | 80 | 0 | 100 | NA | 0 |
|  | 2015 | 23 | 77 | 0 | 100 | NA | 0 |
|  | 2016 | 22 | 78 | 1 | 99 | NA | 0 |
|  | 2017 | NA | NA | NA | NA | NA | 0 |
| **India** | 2008 | NA | NA | NA | NA | NA | 0 |
|  | 2009 | NA | NA | NA | NA | NA | 0 |
|  | 2010 | NA | NA | NA | NA | NA | 1 |
|  | 2011 | NA | NA | NA | NA | NA | 0 |
|  | 2012  Interoperability is allowed at POS but sub-agents still can only work with one bank | NA | NA | NA | NA | NA | 4 |
|  | 2013  Starting 2010, agents may work for more than one bank, but sub-agents can only work for one | 47 | 53 | 0 | 100 | NA | 4 |
|  | 2014 | 37 | 63 | 0 | 100 | NA | 1 |
|  | 2015 | 49 | 51 | 1 | 99 | NA | 1 |
|  | 2016 | 30 | 70 | 1 | 99 | NA | 0 |
|  | 2017 | NA | NA | NA | NA | NA | 0 |
| **Pakistan** | 2009 | NA | NA | NA | NA | NA | 1 |
|  | 2010 | NA | NA | NA | NA | NA | 1 |
|  | 2011 | NA | NA | NA | NA | NA | 0 |
|  | 2012 | NA | NA | NA | NA | NA | 2 |
|  | 2013 | 8 | 92 | 7 | 93 | NA | 1 |
|  | 2014 | 7 | 93 | 8 | 92 | NA | 0 |
|  | 2015 | 8 | 92 | 9 | 91 | NA | 0 |
|  | 2016  Agents can work for multiple FIs as long as they have separate contracts with each. | 9 | 91 | 9 | 91 | NA | 0 |
|  | 2017 | NA | NA | NA | NA | NA | 0 |
| **Kenya** | 2011 | NA | NA | NA | NA | 17.41  (Jan-Mar) | 2 |
|  | 2012 | NA | NA | NA | NA | 18.99  (Mar) | 0 |
|  | 2013  Starting 2010, exclusivity is not allowed between institutions and agents. | 70 | 30 | 76 | 24 | 23.27  (Mar) | 0 |
|  | 2014  Contract between payment service providers and agents cannot be exclusive. | 65 | 35 | 73 | 27 | 26.25  (Mar) | 1 |
|  | 2015 | 69 | 31 | 79 | 21 | 26.79  (Mar) | 0 |
|  | 2016 | 68 | 32 | 81 | 19 | 24.83  (Mar) | 0 |
|  | 2017 | NA | NA | NA | NA | 39.15  (Jan-Mar) | 0 |
|  | 2018 | NA | NA | NA | NA | NA | 1 |
| **Nigeria** | 2011 | NA | NA | NA | NA | NA | 9 |
|  | 2012 | NA | NA | NA | NA | NA | 7 |
|  | 2013  Exclusivity between financial institutions and agents are prohibited. | 38 | 62 | 0 | 100 | NA | 0 |
|  | 2014 | 40 | 60 | 1 | 99 | NA | 2 |
|  | 2015 | 36 | 64 | 1 | 99 | NA | 0 |
|  | 2016  Anyone violating exclusivity may be suspended for a minimum of 1 month. | 32 | 68 | 2 | 98 | NA | 0 |
|  | 2017 | NA | NA | NA | NA | NA | 0 |
| **Tanzania** | 2011 | NA | NA | NA | NA | NA | 0 |
|  | 2012 | NA | NA | NA | NA | NA | 1 |
|  | 2013  Contract between agent and banking institution cannot be exclusive | 47 | 53 | 48 | 52 | NA | 0 |
|  | 2014 | 40 | 60 | 44 | 56 | NA | 1 |
|  | 2015  Agency agreement should provide for non-exclusive use of the agent. | 62 | 38 | 63 | 37 | NA | 0 |
|  | 2016  Later in 2017, it is re-stated that a contract between an agent and a bank or FI cannot be exclusive. | 54 | 46 | 61 | 39 | 16.54  (Mar) | 1 |
|  | 2017 | NA | NA | NA | NA | 19.23  (Mar) | 0 |
| **Uganda** | 2009 | NA | NA | NA | NA | NA | 2 |
|  | 2010 | NA | NA | NA | NA | NA | 1 |
|  | 2011 | NA | NA | NA | NA | NA | 0 |
|  | 2012 | NA | NA | NA | NA | NA | 1 |
|  | 2013  The agent agreement between a mobile service provider and an agent cannot be exclusive. | 33 | 67 | 43 | 57 | NA | 2 |
|  | 2014 | 37 | 63 | 43 | 57 | NA | 1 |
|  | 2015 | 38 | 62 | 47 | 53 | NA | 0 |
|  | 2016 | 39 | 61 | 53 | 47 | NA | 1 |
|  | 2017  An agreement between a financial institution and an agent shall not be exclusive. | NA | NA | NA | NA | NA | 0 |

*Sources:*Data about the percentage of people who are and are not digitally included and the percentage of people who are and are not mobile money users come from Financial Inclusion Insights (2018). Data about the number of mobile money transfer subscriptions for Kenya and Tanzania were retrieved from the each country’s communications authorities’ quarterly reports (Communications Authority of Kenya; Tanzanian Communications Regulatory Authority). Data about the number of mobile money deployments come from GSMA (2018).

**References**

Bourreau, M. & Valetti, T. (2016). Competition and Interoperability in Mobile Money Platform Markets: What Works and What Doesn’t? Retrieved 22 May 2018 from <https://papers.ssrn.com/sol3/papers.cfm?abstract_id=2763624>

Castri S. (2013). Mobile Money: Enabling Regulatory Solutions. Retrieved 22 May 2018 from <https://papers.ssrn.com/sol3/papers.cfm?abstract_id=2302726>

Communications Authority of Kenya. Statistics. Retrieved 1 May 2018 from <http://ca.go.ke/index.php/statistics>

Financial Inclusion Insights. (2018). Data Finder. Retrieved 22 May 2018 from <http://finclusion.org/data_fiinder/>

GSMA. (2018). Mobile Money Metrics. Retrieved 7 June 2018 from <https://www.gsma.com/mobilemoneymetrics/#LatAmCar?y=2011?v=availability?g=regions>

Houpis, G. & Bellis, J. (2007). Competition issues in the development of m-transaction schemes. Vodafone Policy Paper no. 6. Retrieved 22 May 2018 from <https://www.vodafone.com/content/dam/vodafone/about/public_policy/policy_papers/public_policy_series_6.pdf>

Leishman, P. (2010). GSMA Launches Mobile Money Deployment Tracker. GSMA. Retrieved 25 May 2018 from <https://www.gsma.com/mobilefordevelopment/programme/mobile-money/gsma-launches-mobile-money-deployment-tracker/>

Mazer, R., Pillai R., & Staschen S. (2016). Agents for Everyone: Removing Agent Exclusivity in Kenya & Uganda. CGAP. Retrieved 22 May 2018 from <http://www.cgap.org/blog/agents-everyone-removing-agent-exclusivity-kenya-uganda>

Muthiora, B. (2015). Enabling Mobile Money Policies in Kenya. GSMA. Retrieved 22 May 2018 from <https://www.itu.int/en/ITU-T/focusgroups/dfs/Documents/2015_MMU_Enabling-Mobile-Money-Policies-in-Kenya.pdf>

Tanzanian Communications Regulatory Authority. Quarterly Communications Statistics. Retrieved 1 May 2018 from <https://www.tcra.go.tz/index.php/quarterly-telecommunications-statistics>

Tarazi, M. & Breloff, P. (2011). CGAP Regulating Banking Agents. CGAP. Retrieved 22 May 2018 from <https://www.cgap.org/sites/default/files/CGAP-Focus-Note-Regulating-Banking-Agents-Mar-2011.pdf>
