# Supplementary material for: Mobile money and branchless banking regulations affecting cash-in, cash-out networks in low- and middle-income countries
Source: Gates Open Res. 2018 Nov 28;2:64. [Version 1] doi: 10.12688/gatesopenres.12876.1 (PMC6610045; doi:10.12688/gatesopenres.12876.1)
Supplement: Supplementary file 6 [file gatesopenres-2-13965-s0005.tgz › 5f6e528f-852a-4365-86fd-ec44ece91e7d_Supplementary_File_6_Opportunties_for_CICO_Network_and_Agent_Expansion.docx]

**Supplementary File 6: Opportunities for CICO Network and Agent Expansion**

This addendum provides a brief summary of regulations and literature discussing cash-in cash-out (CICO) network and agent expansion. Through our review of recent regulations, publications, and online reports and websites we did not find any examples of companies using or planning to use subsidies to expand agent and CICO networks. Instead, much of the literature discusses the opportunities that existing banks, especially those with greater amounts of resources, have to support agent expansion either through expanding individual agent capacity or through contributing to the development of appropriate regulations.

Broadening CICO networks via branchless banking may extend financial opportunities to low-income and rural communities as branchless access points are often offered through existing infrastructure (e.g., retailers and other trusted intermediaries) that partner with banks or mobile network operators (MNOs) (Lyman, Ivatory, & Staschen, 2006; Radcliffe & Voorhies, 2012; Maurer, Nelms, & Rea, 2013). As seen in Table S6a, access to components of CICO networks, including Automated Teller Machines (ATMs), agents, and commercial bank branches vary widely by country. For example: in Indonesia there are approximately 53 ATMs per 100,000 adults while Uganda has approximately four; there are approximately 918 registered mobile money agent outlets per 100,000 adults in Tanzania but only 21 in Nigeria; and there are approximately 18 commercial bank branches per 100,000 adults in Indonesia compared with only three branches per 100,000 adults in Tanzania.

**Table S6a. Distribution of cash-in, cash-out (CICO) access points by country.**

| **Country** | **ATMs** | | | **Mobile Money Agent Outlets (Active)** | | | **Mobile Money Agent Outlets (Registered)** | | | **Commercial Bank Branches** | |
| --- | --- | --- | --- | --- | --- | --- | --- | --- | --- | --- | --- |
|  | *Per 1,000 km2* | *Per 100,000 adults* | *Per 1,000 km2* | | *Per 100,000 adults* | *Per 1,000 km2* | | *Per 100,000 adults* | *Per 1,000 km2* | | *Per 100,000 adults* |
| Bangladesh | 59.84 | 6.84 | 1870.53 | | 214.01 | 4731.61 | | 541.35 | 73.17 | | 8.37 |
| Indonesia | 54.81 | 53.31 |  | |  |  | |  | 18.25 | | 17.75 |
| India | 61.88 | 19.70 |  | |  |  | |  | 42.54 | | 13.54 |
| Pakistan | 14.00 | 8.77 | 301.78 | | 189.04 | 391.53 | | 245.26 | 15.99 | | 10.02 |
| Kenya | 4.78 | 9.81 |  | |  | 252.92 | | 519.54 | 2.75 | | 5.65 |
| Nigeria | 18.01 | 16.20 |  | |  | 23.15 | | 20.82 | 6.02 | | 5.42 |
| Tanzania | 2.00 | 6.00 |  | |  | 305.91 | | 917.62 | 0.82 | | 2.46 |
| Uganda | 3.08 | 4.44 |  | |  | 545.87 | | 526.65 | 3.01 | | 2.90 |

Source: International Monetary Fund (IMF) Financial Access Survey, 2015.

As discussed in Lyman, Ivatory, & Staschen (2006), Radcliffe & Voorhies (2012), Maurer, Nelms, & Rea (2013), access to CICO networks in rural and remote areas may provide greater opportunities for unbanked and underserved populations to engage in banking and CICO activities. In order to promote access to CICO networks, some countries have developed regulatory language that requires or encourages banks and financial institutions to consider a ratio of rural to urban distribution when establishing access points. We found regulatory documents specifically addressing CICO outlets in the following countries:

*Bangladesh*

- *Prudential Guidelines for Agent Banking Operation in Bangladesh*(2004) requires that banks maintain a minimum ratio of 3:1 for rural to urban agent outlets. This ratio was updated in 2017 with the *Guidance Note for Approval and Operation of Agent Banking Activities of Banks*, stating that the ratio must be 2:1 rural to urban.

*India*

- The *Guidelines for Licensing of “Payments Banks”* (2014) calls for payment banks to establish locations in under-banked states, and to establish business correspondents, ATMs, and other CICO network institutions in remote areas (with at least 25 percent of all physical access points in rural centers).
- The *Financial Inclusion by Extension of Banking Services – Use of Business Correspondents (BCs)* (2010) also requires that retail outlets and sub-agents of Business Correspondents must be located no more than 30 km from the base branch in rural, semi-urban, and urban areas; and no more than 5 km in metropolitan centers. However this regulation states that distance requirements may be modified for proposals to expand outlets to under-banked areas.

*Indonesia*

- The Bank of Indonesia recognizes the difficulty of reaching remote and rural unbanked populations, and therefore encourages banks and MNOs to partner with a wide range of institutions to provide branchless banking services (Stapleton, 2013). Spencer et al. (2017) state that Indonesia’s Financial Services Authority (OJK) intends to specify the ratio of rural to urban agents that must be met when banks propose new agent locations.

*Tanzania*

- Di Castri & Gidvani (2014) state that the Bank of Tanzania requires that agents must have at least five outlets and two of these must be located within rural areas.

In addition to country regulations, some businesses, Non-Governmental Organizations, and research institutions have noted the importance of expanding agent and CICO networks to underserved areas and populations. In collaboration with the Myanmar Development Resource Institute’s Centre for Economic and Social Development (MDRI-CESD), MasterCard finds that reducing Myanmar’s reliance on cash and increasing the population’s familiarity with financial services are both keys to raising countrywide income levels (Valenti, Galloway, & Pwint, 2015). Furthermore, MasterCard has proposed using its expertise to build the capacity of domestic banks and consult with governments to establish new market access points, provide new services, and expand payment structures (*Ibid.*). In another collaboration with MasterCard, Mori & Zimmer (2015) of the Fletcher School at Tufts University assert that while agent network expansion is highly reliant upon an enabling regulatory environment, businesses can work with governments to support the development of regulations that are best suited to the needs of businesses, government, and consumers; businesses for example could share what type of products are most often adopted by consumers so that government can ensure regulations support rather than inhibit the success of these products.

Cheston et al. (2016) of the Center for Financial Inclusion emphasize the importance of agent networks in increasing opportunities to serve unbanked customers, and notes that banks with high profit margins are best prepared to expand these networks. Finally, Peake (2012) argues that in addition to expanding the number of agent outlets, financial institutions must also support the expansion of individual agent capacity to provide the appropriate types and quantities of services; for example, Peake notes that rural agents typically provide more cash-out than cash-in services and therefore may need support from financial institutions to maintain an appropriate amount of liquidity.

**References**

Di Castri, S. & Gidvani, L. (2014). Enabling Mobile Money Policies in Tanzania. GSMA. Retrieved from <https://www.gsma.com/mobilefordevelopment/wp-content/uploads/2014/03/Tanzania-Enabling-Mobile-Money-Policies.pdf>

Cheston, S., Conde, T., Bykere, A., & Rhyne, E. (2016). The Business of Financial Inclusion: Insights from Banks in Emerging Markets. Institute of International Finance & Accion Center for Financial Inclusion. Retrieved from <http://www.centerforfinancialinclusion.org/storage/documents/IIF_CFI_Report_FINAL.pdf>

Lyman, T., Ivatury, G., & Staschen, S. (2006). Use of Agents in Branchless Banking for the Poor: Rewards, Risks, and Regulation. CGAP, Focus Note Number 38. Retrieved 10 November 2017 from <https://www.cgap.org/sites/default/files/CGAP-Focus-Notes-Use-of-Agents-in-Branchless-Banking-for-the-Poor-Rewards-Risks-and-Regulation-Oct-2006.pdf>

Maurer, B., Nelms T., & Rea S. (2013). ‘Bridges to Cash’: Channeling Agency in Mobile Money. *Journal of the Royal Anthropological Institute*, 19(1), 52-74.

Mori, M. & Zimmer, T. (2015). Mobilizing Banking for Indonesia’s Poor. Innovations: Technology, Governance, Globalization, 10(1-2), pp. 95-124. Retrieved from <http://fletcher.tufts.edu/~/media/Fletcher/Microsites/IBGC/Student%20Research/Mobilizing%20Banking%20for%20Indonesias%20Poor.pdf>

Peake, C. (2012). New Frontiers: Launching Digital Financial Services in Rural Areas. The 2012 Brookings Blum Roundtable Policy Briefs. Retrieved from <https://www.brookings.edu/wp-content/uploads/2016/06/10-new-frontiers-peake.pdf>

Radcliffe, D. & Voorhies, R. (2012). A Digital Pathway to Financial Inclusion. The Bill & Melinda Gates Foundation. Retrieved 1 December 2017 from <https://papers.ssrn.com/sol3/papers.cfm?abstract_id=2186926>

Stapleton, T. (2013). Unlocking the Transformative Potential of Branchless Banking in Indonesia. Bulletin of Indonesian Economic Studies, 49(3), pp. 355-380. Retrieved from <https://doi.org/10.1080/00074918.2013.850633>

Spencer, S., Mendoza, J., Amrullah, E., Djauhari, A., Dwipuspita, A., & Nydiasari, R. (2017). Digital Financial Services in Indonesia: An Overview of the USAID e-MITRA Project & Efforts to Advance Digital Financial Services in Indonesia. USAID; NetHope; Global Broadband and Innovations; & e-MITRA. Retrieved from <https://solutionscenter.nethope.org/assets/collaterals/MFS_eMITRAIndonesia-December20151.pdf>

Valenti, A., Galloway, T., & Pwint, M. (2015). Cash in Context: Uncovering Financial Services in Myanmar. MasterCard & Myanmar Development Resource Institute’s Centre for Economic and Social Development (MDRI-CESD). Retrieved from <https://mastercardcenter.org/wp-content/uploads/2015/01/Cash-in-Context-Uncovering-Financial-Services-in-Myanmar.pdf>
