# Supplementary material for: Mobile money and branchless banking regulations affecting cash-in, cash-out networks in low- and middle-income countries
Source: Gates Open Res. 2018 Nov 28;2:64. [Version 1] doi: 10.12688/gatesopenres.12876.1 (PMC6610045; doi:10.12688/gatesopenres.12876.1)
Supplement: Peer review history

# Peer review: Reviewer response for version 1

**DOI:** 10.21956/gatesopenres.13965.r27456

**Published:** 2019-07-02

**Authors:** Erwin A. Alampay

**Affiliations:** 1 National College of Public Administration and Governance, University of the Philippines Diliman, Quezon City, Philippines

The study by Reynolds *et.al*. is really very valuable. It almost reads like a Systematic Research, although it is not as rigorous in terms of being exhaustive in covering various academic databases. While it explains how the filtering of documents down to 31 were done, it did not explain if there was cross validation among the authors which to remove/filter and which to keep.

 What the authors' need to explain further is the logic of how the 8 countries were selected for the regulatory aspect. However, as far as the paper was concerned, the literature on impact included actually went beyond the 8, which made the paper even more relevant to the field.

 The authors might want to briefly define DFS and what it includes.

 Even though the final set of documents had its limitations, the presentation of anecdotal or qualitative impact was much appreciated. It is understandable given the difficulty of actually designing RCTs in this area.

 Figure 1 is a little difficult to comprehend, save for the argument that many of the regulations come in clusters. It does not explain why in some years multiple similar colors occur. Does this mean multiple types of regulation under the same policy?

 In page 9, the trends in caps/fees. Authors might want to elaborate on why these is the policy? Is also AMLA related?

 All in all, I really enjoyed the paper and is a very important contribution to the field.

I confirm that I have read this submission and believe that I have an appropriate level of expertise to confirm that it is of an acceptable scientific standard.

# Peer review: Reviewer response for version 1

**DOI:** 10.21956/gatesopenres.13965.r26955

**Published:** 2019-03-13

**Authors:** Olayinka David-West, Olubanjo Adetunji

**Affiliations:** 1 Lagos Business School, Pan-Atlantic University, Lagos, Nigeria

The paper is well written and provides a detailed overview of the published works on CICO regulations and the impact of regulations on CICO activities. While the study offers a rich literature review, it also highlights the lack of CICO research in sub-Saharan Africa. 

 Regarding the citation of the Intermedia FII date, the authors report the 2015 datasets that may be a little misleading since more recent data sets are available and progress has been made in some of the reported markets, e.g. Tanzania.

 Some other observations:

- Table 1: Customer identification requirements in Nigeria – “PINS” should read “PINs”.
- Results section: The “Trends in business channel requirements” paragraph overly discusses the role of banks and CICO agents even though in some markets the authors identify non-bank actors in the ecosystem.
- Results section: The authors cite Makulilo, 2015 in the paragraph on impacts of business channel requirements, stating "In Tanzania, Nigeria, and Liberia, banks are responsible for overseeing all non-bank mobile money services and are responsible for approving non-bank entities before they can provide mobile money services". This is not true in Nigeria where banks are not responsible for non-bank mobile money services. 
- The authors could also provide some managerial implications as well as some of the study limitations and opine further research opportunities. 

Below are additional comments from my colleague, Dr Olubanjo Adetunji:

 The authors did not discuss how they arrived at the four types of regulations they used to code the 127 regulatory documents found (page 4). I think how they arrived at these four types of regulations should be discussed early in the paper. The authors only hinted on page 10 that the "...categories of regulations identified in our background review of regulations...". The four categories of regulation form the basis on which the documents analyzed in the paper are based. How they arrived at the categories should first be discussed.

We confirm that we have read this submission and believe that we have an appropriate level of expertise to confirm that it is of an acceptable scientific standard.
